# Activated PI3Kδ syndrome, an immunodeficiency disorder, leads to sensorimotor deficits recapitulated in a murine model

**DOI:** 10.1016/j.bbih.2021.100377

**Published:** 2021-10-19

**Authors:** Ines Serra, Olivia R. Manusama, Fabian M.P. Kaiser, Izi Izumi Floriano, Lucas Wahl, Christian van der Zalm, Hanna IJspeert, P. Martin van Hagen, Nico J.M. van Beveren, Sandra M. Arend, Klaus Okkenhaug, Johan J.M. Pel, Virgil A.S.H. Dalm, Aleksandra Badura

**Affiliations:** a Department of Neuroscience, Erasmus MC, Rotterdam, the Netherlands; b Department of Immunology, Erasmus MC, Rotterdam, the Netherlands; c Department of Pediatrics, Erasmus MC, Rotterdam, the Netherlands; d Division of Clinical Immunology, Department of Internal Medicine, Erasmus MC, Rotterdam, the Netherlands; e Department of Psychiatry, Erasmus MC, Rotterdam, the Netherlands; f Department of Infectious Diseases, Leiden University Medical Center, Leiden, the Netherlands; g Department of Pathology, University of Cambridge, Cambridge, United Kingdom; h Academic Center for Rare Immunological Diseases (RIDC), Erasmus MC, Rotterdam, the Netherlands

**Keywords:** Primary immunodeficiency, PID, PIK3CD, APDS, ASD, Mouse

## Abstract

The phosphoinositide-3-kinase (PI3K) family plays a major role in cell signaling and is predominant in leukocytes. Gain-of-function (GOF) mutations in the *PIK3CD* gene lead to the development of activated PI3Kδ syndrome (APDS), a rare primary immunodeficiency disorder. A subset of APDS patients also displays neurodevelopmental delay symptoms, suggesting a potential role of *PIK3CD* in cognitive and behavioural function. However, the extent and nature of the neurodevelopmental deficits has not been previously quantified. Here, we assessed the cognitive functions of two APDS patients, and investigated the causal role of the *PIK3CD* GOF mutation in neurological deficits using a murine model of this disease. We used p110δ^E1020K^ knock-in mice, harbouring the most common APDS mutation in patients. We found that APDS patients present with visuomotor deficits, exacerbated by autism spectrum disorder comorbidity, whereas p110δ^E1020K^ mice exhibited impairments in motor behaviour, learning and repetitive behaviour patterning. Our data indicate that *PIK3CD* GOF mutations increase the risk for neurodevelopmental deficits, supporting previous findings on the interplay between the nervous and the immune system. Further, our results validate the knock-in mouse model, and offer an objective assessment tool for patients that could be incorporated in diagnosis and in the evaluation of treatments.

## 1 Introduction

Primary immunodeficiencies (PID) encompass a group of heterogeneous, mostly inheritable, disorders that affect distinct components of the immune system (Gruber and Bogunovic, 2020; Thaventhiran et al., 2020). Common manifestations of PID include increased susceptibility to infection, autoimmune disease, auto-inflammatory complications and malignancies, ultimately leading to increased morbidity and mortality rates (Amaya-Uribe et al., 2019; Bethune et al., 2019; Odnoletkova et al., 2018; Abolhassani et al., 2018). Activated PI3K delta (PI3Kδ) syndrome (APDS) is a rare monogenic PID, caused by heterozygous mutations in either the *PIK3CD* or *PIK3R1* genes, encoding the p110δ catalytic subunit or the p85α regulatory subunit of PI3Kδ, respectively (Jamee et al., 2019). The most commonly detected variants in APDS patients are the E1021K substitution in p110δ, leading to APDS1, and the 434–475 deletion in p85α, resulting in APDS2 (Coulter et al., 2017; Elkaim et al., 2016). Both mutations lead to gain-of-function (GOF) of PI3Kδ and overactivation of the downstream AKT/mTOR cascade (Dornan et al., 2017; Stark et al., 2018; Nieuwenhuis et al., 2020; Wang et al., 2018). In the immune system, PI3Kδ GOF leads to skewed B cell populations towards a transitional phenotype, decreased numbers of naïve T cells and increased senescent T cells, resulting in impaired vaccine responses and overall immune dysfunction (Stark et al., 2018; Thauland et al., 2019; Wentink et al., 2017). As a consequence, APDS patients present with recurrent infections, lymphoproliferation, autoinflammatory disease and lymphoma (Coulter et al., 2017; Elkaim et al., 2016).

Although predominantly expressed in peripheral blood mononuclear cells (Chantry et al., 1997), PI3Kδ is also detected in murine (Nieuwenhuis et al., 2020; Eickholt et al., 2007) and human (Nieuwenhuis et al., 2020; Hood et al., 2019) brain tissue. In the central nervous system (CNS), the PI3K/AKT/mTOR axis has been shown to play a crucial role in neuronal differentiation and migration (Tee et al., 2016; Jossin and Goffinet, 2007). Accordingly, mutations along this pathway have been commonly associated with neurodevelopmental and neuropsychiatric disorders (Costa-Mattioli and Monteggia, 2013). Although few studies have focused on the specific role of distinct PI3K isoforms in the CNS, PI3Kδ has been proposed to regulate soma size, dendritic complexity and spine number (Nieuwenhuis et al., 2020; Hood et al., 2020; Schmidt et al., 2014), suggesting a contributing role in neuronal development. Notably, 19–31% of APDS patients were reported to exhibit neurodevelopmental delay (Coulter et al., 2017; Elkaim et al., 2016). However, the lack of systematic cognitive evaluation in these reports hinders the quantitative study of PI3Kδ on neurological function. Nonetheless, this putative behavioural role of PI3Kδ is further implied by the report of increased p110δ expression in a person with autism spectrum disorder (ASD) (Poopal et al., 2016).

In this work, we investigated the role of PI3Kδ in motor and cognitive behaviour. We describe two related APDS patients and report, for the first time, a case of APDS-associated ASD. Both APDS patients presented with deficits in visuomotor integration, particularly in tasks requiring response inhibition and memory. The ASD phenotype diagnosed in one of the patients was accompanied by more severe visuomotor impairments. Additionally, we conducted an extensive battery of behavioural tests in an APDS mouse model (Stark et al., 2018), and show that p110δ^E1020K^ mice present with changes in locomotion, learning and repetitive behaviour patterning, consistent with ASD-like phenotype. Taken together, our data suggest that PI3Kδ GOF increases the risk of atypical behavioural development, supporting previous findings on the interplay between the CNS and the immune system.

## 2 Methods

### 2.1 Patients

We describe two APDS patients (Table 2, Table 3). P1 is regularly followed (by VD) at the Primary Immunodeficiency Center of the Department of Internal Medicine, Division of Clinical Immunology, Erasmus MC (Rotterdam, The Netherlands); P2 is currently under treatment (by SMA) at the outpatient Department of Infectious Diseases of Leiden University Medical Center (Leiden, The Netherlands). Psychiatric assessment was performed (by NB) at the Erasmus MC and included the autism-spectrum quotient (Baron-Cohen et al., 2001). Additional clinical history and data were obtained from medical notes (by OM, VD and SMA).

**Table 1:** **Characteristics of controls.** Age-matched individuals were tested in visuomotor assessment tasks and used as controls for patient 1 or patient 2.

Visuomotor assessment	Control 1			Control 2		
	Age (average, years)	SD	N	Age (average, years)	SD	N
Pro-saccade	24,82	5,04	12	54,00	13,39	11
Anti-saccade	25,00	3,35	14	57,70	8,66	17
Memory saccade	24,84	5,28	11	45,70	7,27	10
Pro-tapping	24,76	3,88	10	56,80	13,11	10
Anti-tapping	24,90	7,06	10	55,96	7,68	28
Memory tapping	25,47	5,67	10	53,62	6,31	16
Trajectory prediction∗	38,30	8,78	10	NA	NA	NA

∗ for the trajectory prediction task, only one control group was used.

**Table 2:** **P1 and P2 clinical characteristics**. SLE, systemic lupus erythematosus; ↓, decreased compared to control age-matched range.

Sex	Male (P1)	Female (P2)
Age (diagnosis)	29 (3.5 years)	56 (childhood)
Mutation	E1021K	E1021K
Response to immunization	↓ *S. pneumonia* (polysaccharide response)	↓ *Influenza* type A and type B
Hepato/splenomegaly	Splenomegaly	Absent
Cytopenia	Leucopenia, thrombocytopenia	None
CT-chest results	Air trapping, no bronchiectasis	Bronchiectasis
Hematological malignancy	No	No
Other comorbidities	Psychomotor developmental delay SLE-like auto-immune disease Recurrent EBV infections Autoimmune hepatitis with liver cirrhosis and portal hypertension	None
Ig therapy	Intravenous Ig replacement therapy: 35 ​g, every 3 weeks	Intravenous Ig replacement therapy: 15 ​g, every 4 weeks
Other relevant treatments	Prednisone: 10 ​mg, once daily Mycophenolate mofetil: 500 ​mg, twice daily Hydroxychloroquine: 200 ​mg, once daily Trimethoprim/sulfamethoxazole: 480 ​mg, once daily	No immunosuppressive medication No prophylactic antibiotics

**Table 3:** **P1 and P2 immunological findings.** Abs, absolute numbers; ↓, decreased compared to control age-matched range.

Patient	P1	P2
Ig at diagnosis (g/L; range (Duda et al., 1973))	**IgG** 0.46 *(4.0-11.0)*, **IgA** 0.45 *(0.1-1.6)*, **IgM** 3.24 *(0.5-1.8)*	*Unknown*
T/B/NK cells at diagnosis (abs ​× ​10^9^/L; range (Gomez et al., 1990))	**T cells** 3.66 *(0.9-4.5)*, **CD4**^**+**^**T cells** 0.51 *(0.5-2.4)*, **CD8**^**+**^**T cells** 3.15 *(0.3-1.6)*, **B cells** 0.26 *(0.2-2.1)*, **NK cells** 0.26 *(0.1-1.0)*	*Unknown*
T/B/NK cells (abs ​× ​10^9^/L; range (Gomez et al., 1990)) [B cell subsets in abs, cells/ul]	***Age 22*** **CD3**^**+**^**T cells** 0.23 *(0.7-2.1);***CD4**^**+**^**T cells** 0.1 *(0.3-1.4)*: Naïve (CD4^+^/CD27^+^/CD45RA^+^) 14.4%, Memory (CD4^+^/CD27^+^CD45RA^−^) 83.6%, Effector Memory (CD4^+^/CD27^+^/CD45RA^+/-^) 2.0%; **CD8**^**+**^**T cells** 0.1 *(0.2-1.2)*: Naïve (CD8^+^CD27^+^CD45RA^+^) 37.7%, Memory (CD8^+^/CD27^+^/CD45RA^−^) 33.2%, Effector Memory (CD8^+^/CD27^-^/CD45RA^+/-^) 29.1%; **CD19**^**+**^**B cells** 0.01 *(0.1-0.5)*: Naïve (IgD^+^/CD27^-^) 7 *(57–447)*, Marginal Zone/Natural effector (IgD^+^/CD27^+^) 1 (9-88), Memory (IgD^−^/CD27^+^) 1 *(13–122)* [IgM^+^ 48% (4-37), IgM^−^ 52%]; **CD16.56**^**+**^**CD3**^**−**^**NK cells** 0.04 *(0.09-0.6)*	***Age 43*** **CD3**^**+**^**T cells** 0.53 *(0.7-2.1)* **CD19**^**+**^**B cells** 0.09 *(0.1-0.5)*: **CD16.56**^**+**^**CD3**^**−**^**NK cells** 0.15 *(0.09-0.6)*
Ig (g/L; range (Duda et al., 1973))	***Age 28*** **IgG** 12.0 *(6.0-12.3)*, **IgA** 0.43 *(0.3-2.0)*, **IgM** 3.24 *(0.5-2.0)*	***Age 55*** **IgG** 16.6 *(6.0-12.3)*, **IgA** 1.49 *(0.3-2.0)*, **IgM** 4.75 *(0.5-2.0)*

### 2.2 Visuomotor coordination and memory assessment

**Fig. 1:**
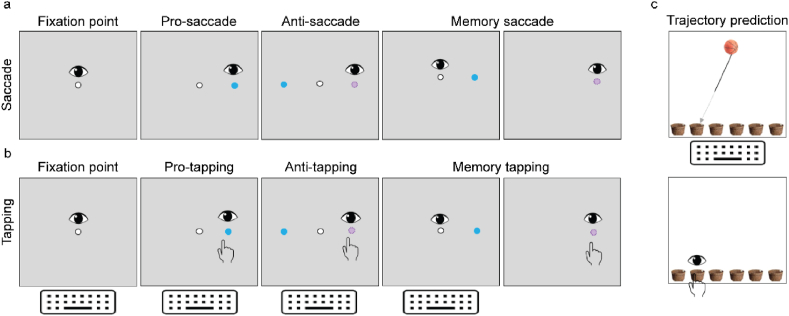
**Visuomotor assessment tasks**. Visual representation of the saccade (a), tapping (b) and trajectory prediction (c) tasks performed by patients P1 and P2, and respective age-matched controls. Pro-tasks involved the execution of reflexive saccades (and tapping) towards a newly appeared target while anti-tasks required a saccade execution (and tapping) to the opposite side of the new target. In memory tasks, subjects waited for target omission to perform a saccade (and tapping). Trajectory prediction tasks involved the execution of a saccade and tapping towards the basket in which a moving ball would be expected to fall. The number and age distribution of control participants per task can be found in Table 1.

An eye-hand coordination measurement setup was used to quantify the interactions between visual, ocular motor, and manual motor systems in both spatial and temporal domains. It consisted of a 21.5″ touchscreen monitor (Wacom DTH-2242, Wacom Corporation, Japan), a remote infrared and screen-based eye-tracker (Tobii Pro X3, Tobii Corporation, Sweden) and a wired keyboard. The eye-tracker, positioned below the touchscreen, recorded eye movements at 120Hz and was connected via an external processing unit to a laptop (DELL Latitude 5590, Dell Technologies, Texas, United States) with an Intel Core i5-8350U processor, 256 ​GB SSD, and 16 ​GB internal RAM to warrant optimal performance and data quality (Pro, 2017). Eye movements with a speed >50°/s were considered saccades. Manual responses were captured by sampling alternating presses and releases of the index finger from the dominant hand, between keyboard and touchscreen. After a short general instruction, each subject was instructed to sit straight in front of the touchscreen. Eye positions were calibrated at approximately 65 ​cm from the touchscreen using a standard calibration procedure. Next, 7 tasks, 3 eye tasks and 4 eye-hand tasks (Fig. 1), were presented on the touch screen in a fixed order (see below). Standard verbal instructions were given prior to each task and each subject was allowed a maximum of three practice trials. These instructions were also written on the screen (in Dutch). The starting position at each trial was fixating a central white dot and, in case of eye-hand tasks, also touching a blue bar at the bottom of the screen with the index finger for 2 ​s. The following 16 trials within each task had to be executed as fast and as accurately as possible. The following tasks were performed:

1. Pro-Saccade: The subject had to fixate on a randomly appearing peripheral dot.
2. Pro-tapping: The subject had to touch a randomly appearing peripheral dot.
3. Anti-Saccade: The participant had to make an eye movement in the opposite direction of a randomly appearing peripheral dot, at either 5, 10, 15 or 20 degrees of the horizontal direction.
4. Anti-Tapping: The participant had to make an eye and hand movement in the opposite direction of a randomly appearing peripheral dot, at either 5, 10, 15 or 20 degrees of the horizontal direction.
5. Memory-Saccade: While fixating the central dot, a peripheral dot appeared for 50 ​ms at a random position. The subject had to fixate on the remembered peripheral dot location after the central dot disappeared.
6. Memory-Tapping: While fixating the central dot, a peripheral dot appeared for 50 ​ms at a random position. The subject had to touch the remembered peripheral dot location after the central dot disappeared.
7. Trajectory Prediction: A ball was dropped in the direction of one of six baskets. Halfway along the trajectory, the ball became invisible and the subject had to touch the basket in which the ball would have fallen.

### 2.3 Mice procedures

Thirty 10 to 13 week-old wild-type (n ​= ​15 WT) and heterozygous p110δ^E1020K^ (n ​= ​15 p110δ^E1020K^) male mice were kindly provided by Dr. Klaus Okkenhaug (University of Cambridge, United Kingdom). These mice harbour an E1020K knock-in mutation in the *Pik3cd* gene expressed in all cells (Stark et al., 2018). Following arrival to the Erasmus MC, mice were acclimated to the facilities for two weeks. Mice were group-housed (3–4 mice per cage, mixed genotypes in the same cage), provided with food and water *ad libitum* and kept on a regular 12h light/dark cycle. After this acclimatization time, mice were handled by the experimenters for three days prior to experiment initiation. Before each experiment, mice were weighed (Supplementary Fig. 3d) and habituated to the testing room for at least 1 ​h. Experimenters were blinded to the genotype of each mouse.

When all behavioural experiments were completed (Supplementary Fig. 1), brain tissue was collected. Mice were injected with an overdose of pentobarbital, transcardially perfused with 0,9% NaCl, and the brain dissected. Tissue was flash frozen and kept at −80 ​°C until used.

### 2.4 Genotyping

Mice were genotyped by amplifying the *Pik3cd* locus from mouse ear DNA using the forward E1020KrecF1 (5′-TCCTCATGGCATCCTTGTCC-3′) primer and the reverse E1020Kflox-recR11 (5′-TGGTCCACCCGTTGACTCAA-3′) primer by PCR. PCR products were run on a 1% agarose gel. The wild-type allele resulted in a 381 bp band and the recombined p110δ^E1020K^ allele resulted in a 436 bp band.

### 2.5 Behavioural box

All mouse behavioural tasks, except the Erasmus ladder, Rotarod and Y-maze, were performed in a behavioural box. This consisted of a 130 ​× ​80 ​× ​80 ​cm wooden box with a door, lined with 6 ​mm high-pressure laminate and foam, with a 10 ​mm Perspex® shelf and standardized white and infrared lights. Metal grooves on the Perspex® shelf assured constant positioning of the testing arenas across experiments. All experiments were recorded with a fixed camera (acA 1300-600 ​gm, Basler AG) positioned above the arenas and operated through the open-source software Bonsai (https://bonsai-rx.org). A frame rate of 25 frames per second (fps) was used for all tests, except for the Grooming assay and the Y-maze, where 30 fps were used. After behavioural testing, video recordings were uploaded to the open-source software OptiMouse (Ben-Shaul, 2017), where each mouse was tracked and measures such as speed and time spent in regions of interest (ROIs) were extracted.

### 2.6 Behavioural tasks

#### 2.6.1 Erasmus ladder

The Erasmus ladder (Noldus, Wageningen, the Netherlands) was used to assess motor performance and learning (Vinueza Veloz et al., 2015), over the course of 5 days, with 42 trials per day. The apparatus consists of a horizontal ladder with 37 high and low rungs between two goal boxes. Each trial starts with the mouse inside a dark goal box and a 9–11s waiting period (Fig. 5a). Three seconds after a light is presented, an air puff encourages the mouse to leave the first box and cross the ladder towards the second box. Once the mouse reaches the second box, a new trial is initiated. After 42 trials, the mouse is returned to its home cage and both ladder and boxes cleaned with 70% ethanol. The frequency of before cue, light cue and air cue exits were automatically recorded by the Erasmus ladder software, together with the percentage of backsteps (steps between the current and previous rung), shorsteps (steps between two adjacent rungs), longsteps (steps between the current and second next rung) and jumps (steps between the current and third or above next rung). Absolute totals per day were calculated off line. The percentage of missteps was also calculated and defined as stepping from, to or in a lower rung (Vinueza Veloz et al., 2015). For one WT and one p110δ^E1020K^ mouse, data from the second day of the Erasmus ladder is missing due to a power failure during acquisition.

#### 2.6.2 Social interaction

Social interaction was evaluated with the three-chamber apparatus as previously described (Badura et al., 2018). This consisted of a 63 ​× ​42.5 ​× ​21 ​cm transparent acrylic arena, equally divided in three chambers, separated by two black-opaque movable partitions. On the day prior to testing, age-matched novel mice, of the same strain but different litter, were habituated to wire cups (8 ​cm diameter, 9.5 ​cm height) for two periods of 10 ​min each. On the following day, a test mouse was first allowed to explore the central chamber of the apparatus for 10 ​min. After this period, the partitions to the right and left chambers were opened, allowing the mouse to explore the full apparatus (*baseline*) during 10 ​min. The test mouse was then guided to and kept in the central chamber, and an empty wire cup and a wire cup with a novel mouse were placed in a pseudo-randomized fashion in the right and left chambers. The partitions were once again opened and the test mouse was allowed to explore the full apparatus for another 10 ​min (*test*). At the end of each test and between animals, chambers, partitions and cups were cleaned with 70% ethanol.

#### 2.6.3 Grooming

The grooming apparatus consisted of a 30 ​× ​30 ​× ​39 ​cm white-opaque Perspex® arena. Mice were placed in the arena and allowed to freely explore for 30 ​min, under dark conditions. At the end of the trial, the arena was cleaned with 70% ethanol and dried before the next mouse was tested. The time and number of grooming events (defined as in (van den Boom et al., 2017) was logged in each recorded video using the open-source software BORIS (Friard and Gamba, 2016).

#### 2.6.4 Elevated-plus maze

To investigate the presence of anxiety-like behaviour, an elevated-plus maze (EPM) with two open and two closed arms was used. Each arm was supported by a 30 ​× ​3 ​cm cylindrical pole and had a 29.5 ​× ​8.5 ​cm white-opaque acrylic base. Closed arms were additionally surrounded by a 20 ​cm high black-opaque acrylic wall. At the beginning of each experiment, the test mouse was placed in the centre of the maze and, after 10 ​min of exploration, returned to its homecage and the maze cleaned with 70% ethanol. The time spent in the closed, open and central areas of the maze, together with the number of entries into each area were calculated.

#### 2.6.5 Open-field

To evaluate spontaneous locomotor activity and speed, mice were tracked under light conditions during the open-field (OF) test, in a 50 ​× ​50 ​× ​35 ​cm white-opaque arena. A test mouse was placed in the centre of the arena and allowed to freely explore for 30 ​min. After test completion, the mouse was returned to its homecage and the arena cleaned with 70% ethanol. Given that anxiogenic behaviour is associated with increased time spent closer to the walls of the OF (Prut and Belzung, 2003), ROIs were defined during video analysis (Fig. 4a) to further determine the time each mouse spent in the inner, outer and corner areas of the OF.

#### 2.6.6 Marble burying

The marble burying (MB) test was used to assess repetitive behaviour and anxiety under light conditions (Sonzogni et al., 2018). A standard 26.6 ​× ​42.5 ​× ​18.5 ​cm cage (Eurostand 1291H-Type III H) was filled with 4 ​cm of bedding (Lignocel® Hygienic Animal Bedding, JRS) and 20 blue glass marbles, in 5 rows of 4 marbles, were set on its surface (Supplementary Figs. 4a and b). A test mouse was carefully placed inside the cage and removed after 30 ​min of exploration. For each mouse, new bedding was used and marbles were cleaned with 70% ethanol.

For the analysis of the surface and number of buried marbles, the open-source programme Fiji was used (Schindelin et al., 2012). The first (before testing) and last (after testing) frames of each acquired video were loaded into Fiji and, after scale adjustment, the *freehand selection* tool was used to manually define a ROI around the visible area of each marble. The difference between the visible area on the first and last frames was used to calculate the buried area for each marble and the sum of differences used to calculate the total buried area for each mouse. The number of buried marbles was also determined and a marble was considered buried if its visible surface was reduced in the last frame by 50% or more (Sonzogni et al., 2018).

#### 2.6.7 Y-maze

Flexible learning was tested over the course of 5 days using the water Y-maze, a 3-armed Y-shaped apparatus of white-opaque acrylic (Fig. 5g). Each arm (20 ​× ​32 ​× ​9 ​cm) has two lateral indentations, 5.5 ​cm from the centre, that fit a 19.5 ​× ​8 ​× ​0.5 ​cm white-opaque acrylic wall used during *forced sessions* (described below). The Y-maze was placed in a dark chamber with fixed black poster-board screens on three sides and a movable black curtain on the fourth side. Above the Y-maze, a fixed camera (Sony PS3 Eye) was used to record all trials and enable tracking of distance swam, speed and body position.

In the beginning of each experimental day, the Y-maze was filled with room-temperature water and white paint (Basic color, 21 white 30081, Creall®), to reduce platform visibility. On day 1 (*Habituation*), each mouse swam freely, without a platform, for 3 consecutive 60 ​s trials, starting from the bottom, then the left and finally the right arm. On day 2 (*Acquisition*), a white-opaque acrylic platform was pseudo-randomly placed in the extremity of the right or left arm. Always starting from the bottom arm, each mouse was placed in the water and allowed to search for the platform over 4 sessions, each with 5 consecutive trials of 40 ​s. On day 3 (*Test*), the hidden platform was kept on the same side as in day 2. Each mouse performed one single session of 5 consecutive trials, with 40 ​s per trial. On day 4 (*Reversal I*), the position of the platform was changed to the opposite side (right or left) of the one defined during *Acquisition* and *Test*. Mice searched for the new location of the platform during 5 sessions, each with 5 consecutive trials of 40 ​s. During session 5, the incorrect arm was blocked (*forced session*), forcing the mouse to eventually swim into the correct arm. On day 5 (*Reversal II*), the protocol used on day 4 was repeated.

Offline manual scoring of correct and incorrect choices was performed for each trial of days 2–5. A correct choice was considered if the mouse reached the hidden platform upon the first turn from the bottom arm into the correct (right or left) arm. Data from mice that did not achieve an 80% correct choice rate during *Test*, was not used for analysis of *Reversal I* and *Reversal II* data, as it was considered that these mice did not effectively learn the location of the platform. Two WT and two p110δ^E1020K^ mice met this exclusion criteria.

After each trial, mice were dried with tissue paper and, after each session, returned to their homecage and placed under a heating lamp. At the end of each day, the Y-maze was emptied, cleaned with tap water and 70% ethanol, and dried.

#### 2.6.8 Rotarod

Motor performance was assessed with the rotarod (Reith et al., 2011). We used a five-day accelerating rod protocol, each with 4 non-consecutive trials per mouse per day. From days 1–4, the speed of the rotating rod was accelerated to 40 ​rpm while, on day 5, maximal speed was increased to 80 ​rpm. The time each mouse stayed on the rotarod was counted by the apparatus’ stopwatch and manually recorded for each trial. Between trials, mice were given a 1 ​h resting period, and the rod and separating walls were cleaned with 70% ethanol and dried with paper towels. A trial was considered finished when the mouse fell off the rod, grabbed the moving rod performing a 360° turn without actually walking on the rod or reached a maximum time of 300 ​s on the rod. The average time per trial across the five days and the total time spent on the rod were calculated.

### 2.7 Western blot

Brain tissue was lysed and homogenized in RIPA Lysis and Extraction Buffer (Thermo Scientific™), supplemented with Halt™ Protease and Phosphatase Inhibitor Cocktail (Thermo Scientific™). Protein concentration was determined with Pierce™ BCA Protein Assay Kit (Thermo Scientific™). Protein lysates were mixed with 4X Laemmli Sample Buffer, supplemented with 2-mercaptoethanol (Bio-Rad Laboratories B.V.) and incubated at 100 ​°C for 6 ​min. Eighty μg (brain and spleen tissue of WT and p110δ^E1020K^ mice) or 40 ​μg (splenocytes of WT and Pik3cd^-^^/-^ mice) of lysate were loaded onto 4–15% Mini-PROTEAN® TGX™ Precast Protein Gels (Bio-Rad Laboratories B.V.). Transfer was performed onto Immobilon®-P PVDF Membranes (Merck KGaA). Membranes were blocked with 5% BSA (Merck KGaA) in TBS (Merck KGaA) for 1 ​h and subsequently incubated with anti-PI3K p110δ (D1Q7R) Rabbit mAb (1:1.000, #34050, Cell Signaling Technology, B.V.) in 5% BSA-TBS with 0,1% Tween 20 (TBS-T) (Merck KGaA) overnight at 4 ​°C. Membranes were washed three times with TBS-T and incubated with IRDye® 800CW Goat anti-Rabbit IgG (1:10.000, H ​+ ​L; LI-COR Biosciences - GmbH) in 5%-BSA-TBS-T for 1 ​h at room temperature. Membranes were washed three times with TBS-T and imaged in an Odyssey® CLx Imaging System. Afterwards, membranes were incubated with GAPDH (D16H11) XP® Rabbit mAb (1:1.000 dilution, #5174, Cell Signaling Technology, B.V.) in 5% BSA-TBS-T overnight at 4 ​°C. Membranes were washed three times with TBS-T and incubated with IRDye® 800CW Goat anti-Rabbit IgG (1:10.000, H ​+ ​L; LI-COR Biosciences - GmbH) in 5%-BSA-TBS-T for 1 ​h at room temperature. Membranes were washed three times with TBS-T and imaged in an Odyssey® CLx Imaging System. Western blots were visualized with Image Studio Lite™ software (LI-COR Biosciences - GmbH).

### 2.8 Linear discriminant analysis

For multivariate analysis, linear discriminant analysis (LDA) was performed to identify the behavioural features that best separate WT and p110δ^E1020K^ genotypes (Machado et al., 2020). LDA is based on an eigenvector optimization problem that searches for a set of lines that maximizes distance and minimizes variance, separating predefined classes (in this case, genotype) (Duda et al., 1973).

$$Set\;of\;eigenvector\;\propto \frac{{\left|\mu_{1}-\mu_{2}\right|}^{2}}{\sigma {\;}_{1}^{2}+\sigma {\;}_{2}^{2}}=\frac{\left|S_{B}\right|}{\left|S_{W}\right|}=S_{W}^{-1}S_{B}$$

Variables that consisted of multiple data points, measured over several sessions (e.g. rotarod data, acquired over the course of 5 days), were reduced to a single value variable by calculating the slope across data points, as this can be interpreted as a learning curve of an animal for a given variable. After pre-processing and validation, LDA was performed with a custom written code. The outcome from the LDA was plotted as LD1 vs LD2, with the contribution of the 10 best variables per LD. All code used to perform the pre-processing steps, validation and LDA is available at https://github.com/BaduraLab/LDA_analysis_2_classes.

#### 2.8.1 Pre-processing

Before conducting the LDA, data were pre-processed to comply with the normality assumption by calculating z-scores (Duda et al., 1973). Z-scores were inspected for every variable and compared with a standard normal distribution. Due to its highly skewed distribution, “Y-maze: reverse II” data were excluded from further analysis. Data points that exceeded 3 scaled median absolute deviations from the median (*isoutlier* function in Matlab) of the corresponding class per variable, were considered outliers and excluded from further analysis. Excluded outliers were interpolated with the mean of their corresponding class per variable (mean interpolation) (Duda et al., 1973; Gomez et al., 1990)**.**

Next, a correlation matrix with all tested behaviours was generated (Supplementary Fig. 6a) to exclude strongly correlated variables. Inspection of the matrix identified *speed* related variables as strongly correlated (r ​≥ ​0.86) with measures of *total distance* of the corresponding experiment. Therefore, speed variables were excluded in this step, while distance variables were kept for further analysis, which resulted in 31 behavioural measures included in the LDA. Finally, we applied the Moore-Pseudo Inverse method to allow inclusion of all variables in the analysis by approximating the inverse of the within variance matrix (Ng et al., 2011). This last step was necessary because one of LDA's criteria is that the total number of variables analysed must be lower or equal to the total number of samples minus the number of classes (Duda et al., 1973).

#### 2.8.2 LDA validation

To validate the results of the LDA, data was shuffled 200.000 by randomizing data labelling. This number was chosen by shuffling a random dataset *N* times until an error margin of under 5% was achieved, based on the concept of a Monte Carlo simulation (Landau and Binder, 2005). With each shuffle, the individual data points were randomly assigned to two equally sized classes. After each round, LDA was performed to investigate the new features’ contributions.

While dominant features can still appear dominant while shuffling, provided that these variables are actually not dependent, exactly equal combinations of contributions were predicted to be low and therefore different from the final LDA results. After shuffling, the first LD1 variable, *Time on ladder (EL)*, appeared 0.21% of the times in 1st place, the second variable, *Light/air ratio (EL)*, was above 18.73% of the times in 2nd place, and the third variable, *Total time (G)*, appeared 1.81% of the times in 3rd place (Supplementary Fig. 6b). The rank sum of the first two features appeared 0.013% of times and the rank sum of the first three appeared 0% of times.

### 2.9 Statistics

For the analysis of patient visuomotor data, a customized MATLAB script (Mathworks, Natick, MA, USA) was used to visually inspect and analyze all the measured trials. Three outcome measures were considered: 1) Performance - percentage of correctly performed trials; 2) Eye Latency (EL) - time between the presentation of a peripheral stimulus and initiation of the primary saccadic eye movement; 3) Hand Latency (HL) - time between the presentation of a peripheral stimulus and the release of the index finger from the keyboard. The control groups, C1 and C2, were age-matched to patients P1 and P2, respectively. The age and number of control patients is presented in Table 1.

For mouse behavioural data, statistical analysis involving hypothesis testing and group comparison was performed with the Graphpad Prism 8 software. Data sets were first tested for the presence of significant outliers using the Grubbs test, and then for the assumption of normality, using the Shapiro-Wilk test and Q-Q plots. When normality was followed, WT and p110δ^E1020K^ groups were compared with a two-tailed *t*-test or a 2-way repeated measures ANOVA, depending on the parameters analysed. When data violated the assumption of normality, a two-tailed Mann-Whitney test was performed instead. A mixed effects model was used in place of repeated measures ANOVA when data points were missing or excluded (outliers). The statistical significance threshold was set at p ​≤ ​0.05. For the analysis of automatically tracked behaviour, body position values were used, except for the “near cup” parameters of the social interaction task. In this case, the nose position was extracted to more accurately represent the interaction between test and novel mice (*sniffing* the novel mouse).

### 2.10 Study approval

Patient P1 had previously been recruited for a longitudinal, multi-centre, cohort study on the causes and clinical manifestations of PID. For this study, approval of the Medical Ethics Committee of the Erasmus University Medical Center Rotterdam had been obtained (MEC, 2013–026). Written informed consent was obtained from patients P1 and P2 according to the Declaration of Helsinki.

All experimental animal procedures were approved a priori by an independent animal ethical committee (DEC-Consult, Soest, The Netherlands), as required by Dutch law and conform to the relevant institutional regulations of the Erasmus MC and Dutch legislation on animal experimentation.

## 3 Results

### 3.1 Immunological profile and neuropsychiatric manifestations of APDS patients

We present a 29-year-old male patient, P1, the second child of non-consanguineous parents of Caucasian descent (P2) (Table 2, Table 3) (Wentink et al., 2017). Since the age of 9 months, P1 suffered from recurrent upper and lower respiratory tract infections and diarrhoea. At the age of 3.5 years, P1 was hospitalized for generalized lymphadenopathy due to EBV infection. He was subsequently diagnosed with common variable immunodeficiency, based on low serum IgG and IgA levels (with elevated IgM levels), and recurrent infectious complications for which intravenous immunoglobulin replacement therapy was initiated. At the age of 7, P1 developed auto-immune complications, including cutaneous manifestations, fever, arthritis, anaemia, thrombocytopenia and hepatosplenomegaly, with positive antinuclear antibody and anti-dsDNA titres, described as systemic lupus erythematosus (SLE)-like disease, for which immunosuppressive therapy was initiated. Other complications included liver cirrhosis due to auto-immune hepatitis with portal hypertension, requiring liver transplantation in December 2020. At age 22, genetic testing revealed a c.3061 ​G ​> ​A mutation in the *PIK3CD* gene, resulting in an E1021K substitution and APDS1 (Angulo et al., 2013; Nunes-Santos et al., 2019).

Next to the immunological phenotype, we also observed neuropsychological deficits in P1. Psychomotor developmental delay was present, as the patient started walking at the age of 2 and speaking at age 2,5. At age 6, ASD was considered and P1 was referred to special needs education. At the age of 9 years, intelligence quotient testing indicated a score of 80. Moreover, P1 showed persistent deficits in social interaction, motor function and a distinct fascination for watches, calendars and dates. P1 was diagnosed with pervasive developmental disorder not otherwise specified at age 10, and re-evaluation in 2020 confirmed the diagnosis of ASD based on psychiatric examination and on the autism-spectrum quotient (Baron-Cohen et al., 2001). To date, P1 requires assistance with tying shoelaces and buttoning his shirts.

Patient 2 (P2), who has been previously described (Wentink et al., 2017), is a non-consanguineous parent from P1. Genetic testing revealed a c.3061 ​G ​> ​A mutation in the *PIK3CD* gene, resulting in the E1021K substitution which was also found in P1. P2 suffered from recurrent upper and lower respiratory tract infections since childhood and was diagnosed with an IgG2 and IgG4 subclass deficiency. She then commenced immunoglobulin replacement therapy and has been on intravenous treatment since. A recent CT-scan showed bronchiectasis. There have been no signs of hepatosplenomegaly nor lymphadenopathy. Currently, her clinical phenotype is relatively mild, with no recurrence of severe infections, no auto-immune complications, no inflammatory disease and no hematological malignancy. She was never diagnosed with a neurodevelopmental condition.

### 3.2 APDS patients present with deficits in visuomotor integration

Previous clinical descriptions of APDS reported the presence of cognitive impairment, developmental delay or speech delay in a number of patients (Coulter et al., 2017; Elkaim et al., 2016). Given the formal diagnosis of ASD in P1, and its association with attention and motor performance (Del Bianco et al., 2020; Choi et al., 2018; Mous et al., 2017), we conducted a series of tests to evaluate visuomotor performance in both patients (Fig. 1a–c).

**Fig. 2:**
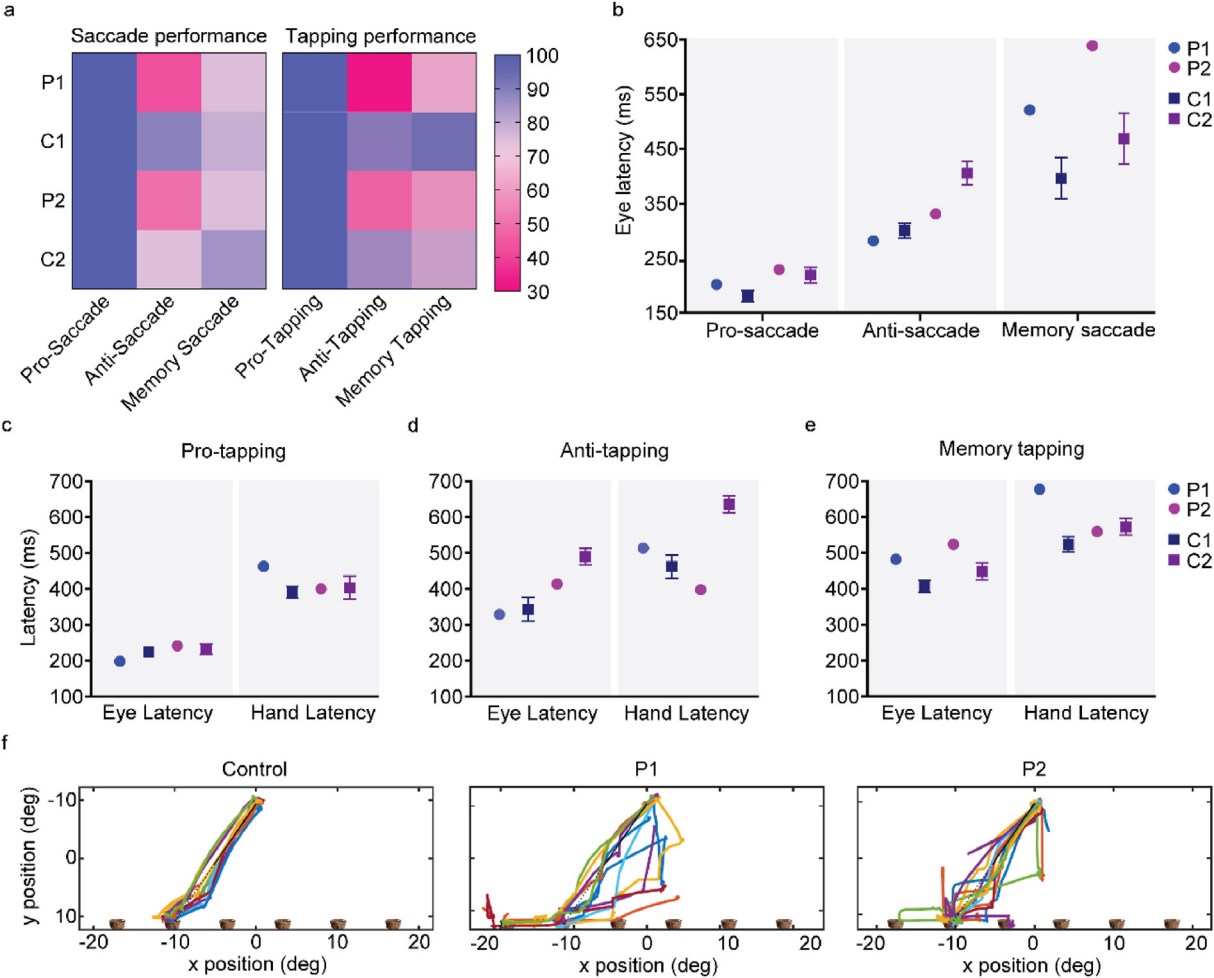
**APDS patients present with intact reflexive saccades but altered integration**. a) Performance in the saccade and tapping tasks is presented as percentage of correct trials. Eye latency for the saccade tasks (b), and eye and hand latency for the tapping tasks (c–e) are presented in ms. f) Representative traces of the eye trajectories performed towards one basket, during the trajectory prediction task. P1, patient 1, P2, patient 2; C1, age-matched controls for patient 1, C2, age-matched controls for patient 2.

Visual reflexive behaviour, primarily driven by parietal eye field and brainstem functions (Müri et al., 1996; Van Der Werf et al., 2009), was intact in both patients, with performance in the pro-saccade test equal between P1, P2 and their respective age-matched controls (performance: 100% for all groups) (Fig. 2a). While pro-tapping performance was also similar for all cohorts (performance: 100% for all groups), P1 exhibited increased hand latency compared to the other groups. Specifically, P1 average latency was over 4.5 SD higher than the age-matched control group (C1) (P1 ​= ​463 ​ms, C1 ​= ​391 ​± ​16 ​ms, P2 ​= ​400 ​ms, C2 ​= ​403 ​± ​33 ​ms) (Fig. 2a, c).

To understand whether this increased hand latency was due to a motor impairment or rather a consequence of increased task complexity, motor command and execution were tested in the trajectory prediction test (Fig. 1c). Both patients exhibited similar latencies in decisive saccades towards the target basket, indicating that the task was correctly understood (P1 ​= ​513 ​ms; P2 ​= ​570 ​ms; C ​= ​539 ​ms). Average hand latency was also similar for both P1 and P2 when compared to control groups, suggesting intact preparation and onset of motor response (P1 ​= ​779 ​ms; P2 ​= ​761 ​ms; C ​= ​767 ​ms). However, while P2 exhibited similar performance to controls, P1 presented with a reduction in the percentage of correct trials (P1 ​= ​78%, P2 ​= ​100%, C ​= ​96%) (Fig. 2f). Moreover, both APDS patients adopted a less systematic strategy to follow the ball's trajectory compared to controls, exhibiting less goal-directed scan paths and more irregular eye gaze (Fig. 2f). These data suggest that, while preparation and onset of motor responses appear to be intact in both patients, increased task speed and complexity likely impairs integration, particularly in P1.

We next tested volitional inhibitory behaviour using the anti-saccade and anti-tapping tests (Fig. 1a and b). Both tests require a suppression of reflexive pro-saccades and engage a complex network of brain regions, including dorsolateral prefrontal cortex, frontal eye fields, and supplementary eye fields, basal ganglia, superior colliculus and cerebellum (Everling and Fischer, 1998; Munoz and Everling, 2004; Pierrot-Deseilligny et al., 2004). The anti-saccade task has been used to characterize cognitive impairments in patients with schizophrenia (Nieman et al., 2007; Kang et al., 2011), dementia (Meyniel et al., 2005), Parkinson's disease (Waldthaler et al., 2019) and cerebellar atrophies (Piu et al., 2019).

In the anti-saccade and anti-tapping tests, both APDS patients underperformed controls (anti-saccade performance: P1 ​= ​43%, C1 ​= ​89%, P2 ​= ​50%, C2 ​= ​74%; anti-tapping performance: P1 ​= ​31%, C1 ​= ​88%, P2 ​= ​50%, C2 ​= ​83%) (Fig. 2a). While patient eye latency was faster when compared to respective controls (P1 ​= ​329 ​ms, C1 ​= ​344 ​± ​33 ​ms, P2 ​= ​414 ​ms, C2 ​= ​490 ​± ​23 ​ms), indicative of frontal inhibition deficits (Muilwijk et al., 2013), P1 hand latency was increased during tapping (P1 ​= ​514 ​ms vs C1 ​= ​462 ​± ​33 ​ms; 1.6 SD difference). P2 presented delayed hand execution time (time between screen bar release to target) (P2 ​= ​992 ​ms vs C2 ​= ​132 ​± ​35 ​ms; > 24.5 SD difference) combined with faster hand latency (P2 ​= ​398 ​ms vs C2 ​= ​636 ​± ​23 ​ms) (Fig. 2b, d). Together, our data show that P1 presents with movement integration deficits while P2, despite better performance to age-matched controls, exhibits delayed movement execution.

To further evaluate integration deficits, both patients performed a spatial memory task requiring both inhibition and memory retrieval (Fig. 1a and b). While performance in the memory-saccade task was similar for all groups (P1 ​= ​75%, C1 ​= ​79%, P2 ​= ​75%, C2 ​= ​85%) (Fig. 2a), both patients exhibited delayed eye latency (P1 ​= ​483 ​ms, C1 ​= ​406 ​± ​17 ​ms; > 4.5 SD difference, P2 ​= ​524 ​ms, C2 ​= ​449 ​± ​24 ​ms; > 3 SD difference) (Fig. 2b). In line with the anti-tapping task, P1 presented with increased hand latency, compared to controls (P1 ​= ​678 ​ms vs C1 ​= ​524 ​± ​21 ​ms; > 7 SD difference) (Fig. 2e), whereas P2 exhibited severely delayed hand execution time (P2 ​= ​919 ​ms vs C2 ​= ​146 ​± ​39 ​ms; > 19.5 SD difference). These results show that, while target location was remembered by both patients, in addition to the aforementioned motor integration deficits, recalling target position was delayed.

### 3.3 PI3Kδ is expressed in adult mouse brain

Our patient data suggested that *PIK3CD* GOF increased the risk of neuropsychiatric dysfunction, supporting previous reports (Coulter et al., 2017; Elkaim et al., 2016). To fully characterize the extent of neurological deficits and establish an animal model to test future pharmacological interventions, we resorted to a heterozygous mouse model of APDS (E1020K knock-in mouse, further referred to as “p110δ^E1020K^ mice”) (Stark et al., 2018), to explore the effects of *Pik3cd* GOF on behaviour.

Prior work in WT mice with a lacZ-*p110δ* reporter indicated the presence of p110*δ* in adult brain, predominantly in the cortex and hippocampus (Eickholt et al., 2007). Supporting these results, we detected an 110 ​kDa band in both WT and p110δ^E1020K^ brain tissue (Supplementary Fig. 2). p110δ was highly expressed in the spleen, as expected due to abundant B cell populations (Clayton et al., 2002). In the brain, we found lower expression levels of p110δ, primarily detected in the cortex, hippocampus and olfactory bulbs (Supplementary Fig. 2).

### 3.4 p110δ^E1020K^*mice exhibit intact gross motor skills but altered locomotion pattern*

**Fig. 3:**
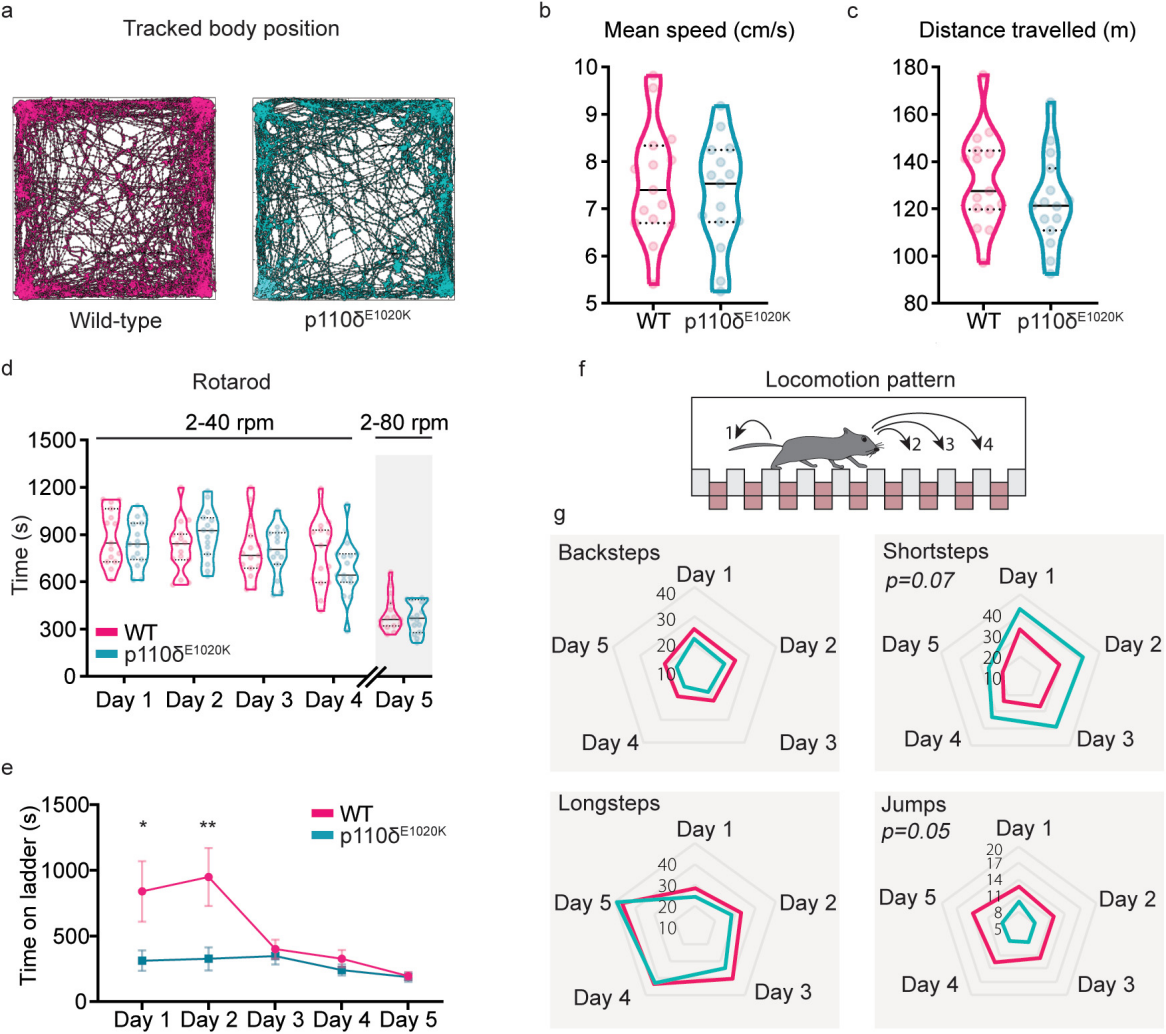
**Slight fine locomotion impairments are caused by the murine E1020K mutation**. a) Example of automatically tracked trajectories showing the body position of a WT and a p110δ^E1020K^ mouse during the 30 min of the OF task. b-c) Quantification of the mean speed (n ​= ​15 per genotype) and total distance travelled (n ​= ​15 WT and 14 p110δ^E1020K^) during the OF task, presented as median and quartiles (2-tailed *t*-test). d) The total time each mouse spent on the rotarod, over the course of 4 trials/day, is presented as median and quartiles (2-way repeated-measures ANOVA, n ​= ​15 per genotype). On the last day, the maximum rod speed was increased to 80 ​rpm. e-g) The Erasmus ladder was used to investigate locomotion pattern. The average time each mouse spent on the ladder, across 42 daily trials, is presented in (e) (2-tailed Mann-Whitney; data presented as mean ​± ​SEM). The distinct step types analysed are schematically represented in (f) and quantified in (g) (Mixed effects model; data is presented as daily mean percentage, n ​= ​15 per genotype). ∗p ​≤ ​0.05, ∗∗p ​≤ ​0.01, ∗∗∗p ​≤ ​0.001.

Having confirmed the expression of PI3Kδ in the brain, we proceeded with behaviour testing. We first assessed motor performance, which is found to be impaired in a number of patients with neurodevelopmental delay, particularly ASD (Bishop and Pangelinan, 2018; Mostofsky et al., 2009). Spontaneous locomotion was tested in the open-field arena (Fig. 3a). Both WT and p110δ^E1020K^ mice moved more during the first 10 ​min of exploration (Supplementary Fig. 3a), with mean speed and distance travelled across the total 30 ​min of testing similar between genotypes (speed: *t*(Vries deet al, 2000) ​= ​0.5494, *p* ​= ​0.59; distance: *t* (Vries deet al, 2000) ​= ​1.234, *p* ​= ​0.22) (Fig. 3b and c). PI3Kδ mutation also did not affect performance on the rotarod test (Fig. 3d) (main effect of genotype, *F*(Gruber and Bogunovic, 2020; Vries deet al, 2000) ​= ​0.1789, *p* ​= ​0.68), indicating that p110δ^E1020K^ mice have no gross motor defects.

**Fig. 4:**
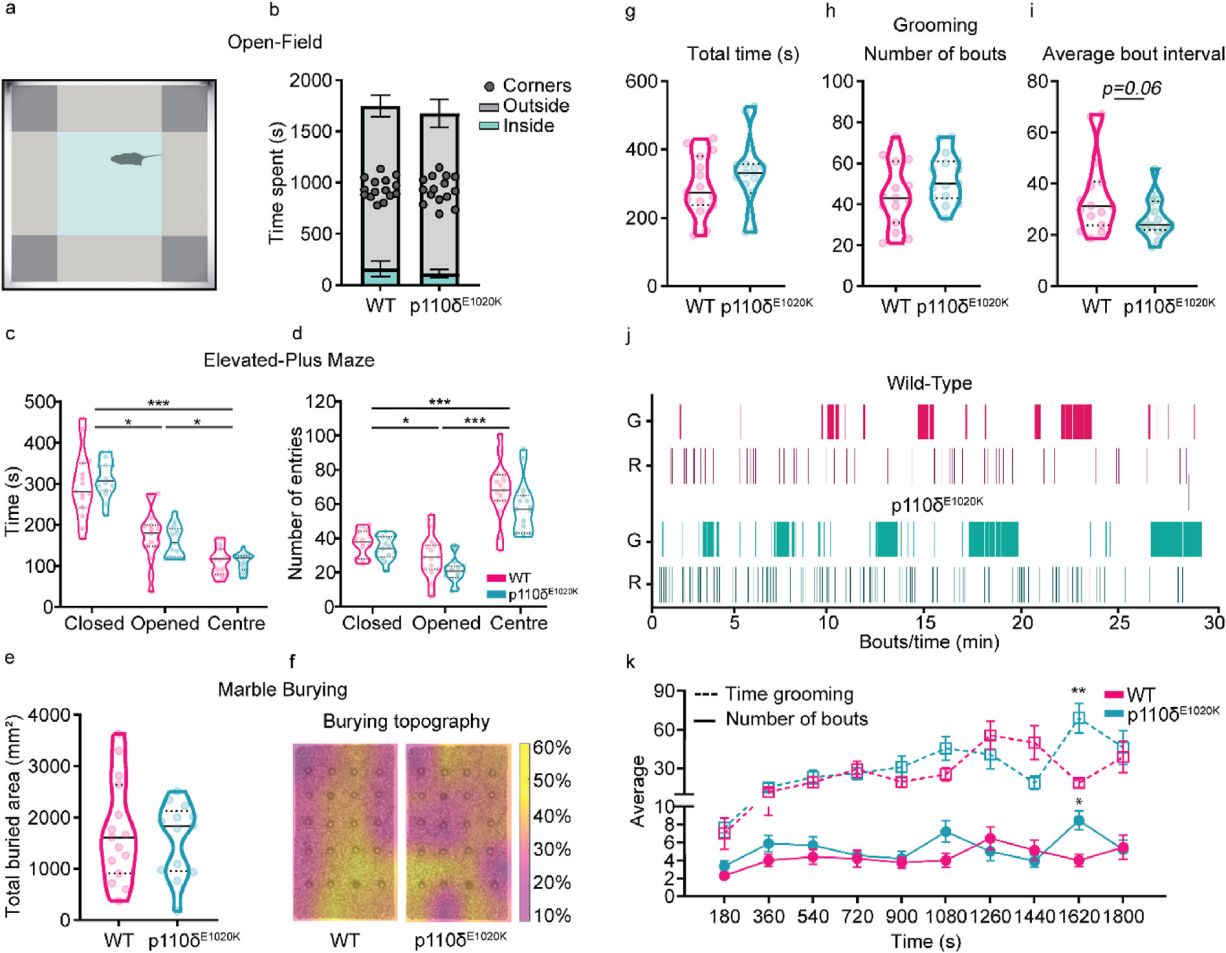
**p110δ**^**E1020K**^**mice exhibit subtle changes in burying and grooming patterns**. a) Representation of the OF arena parcellation into corner, outside and inside areas. b) Total time spent on each OF area (2-tailed Mann-Whitney; data presented as mean ​± ​SD). c-d) Total time spent and number of entries performed in each EPM area (2-way repeated-measures ANOVA; data are presented as median and quartiles). e) Total marble area buried during the MB task (2-tailed *t*-test). f) Marble disposition before the task, superimposed with the average percentage of buried area per marble (n ​= ​15 WT and n ​= ​13 p110δ^E1020K^). g-i) Quantification of the total time spent grooming (g), total number of grooming bouts (h) and the average time interval between grooming bouts (i), during the grooming assay (2-tailed *t*-test; data presented as median and quartiles). j) Representative plot depicting grooming and rearing events for one mouse of each genotype. k) Time-binned plot with the average time spent grooming (dashed line; 2-way repeated-measures ANOVA) and the average number of grooming bouts (full line; mixed effects model) (data are presented as mean ​± ​SEM). G, grooming, R, rearing; ∗p ​≤ ​0.05, ∗∗p ​≤ ​0.01, ∗∗∗p ​≤ ​0.001, n ​= ​15 mice per genotype, expect for e) and f) (see above).

**Fig. 5:**
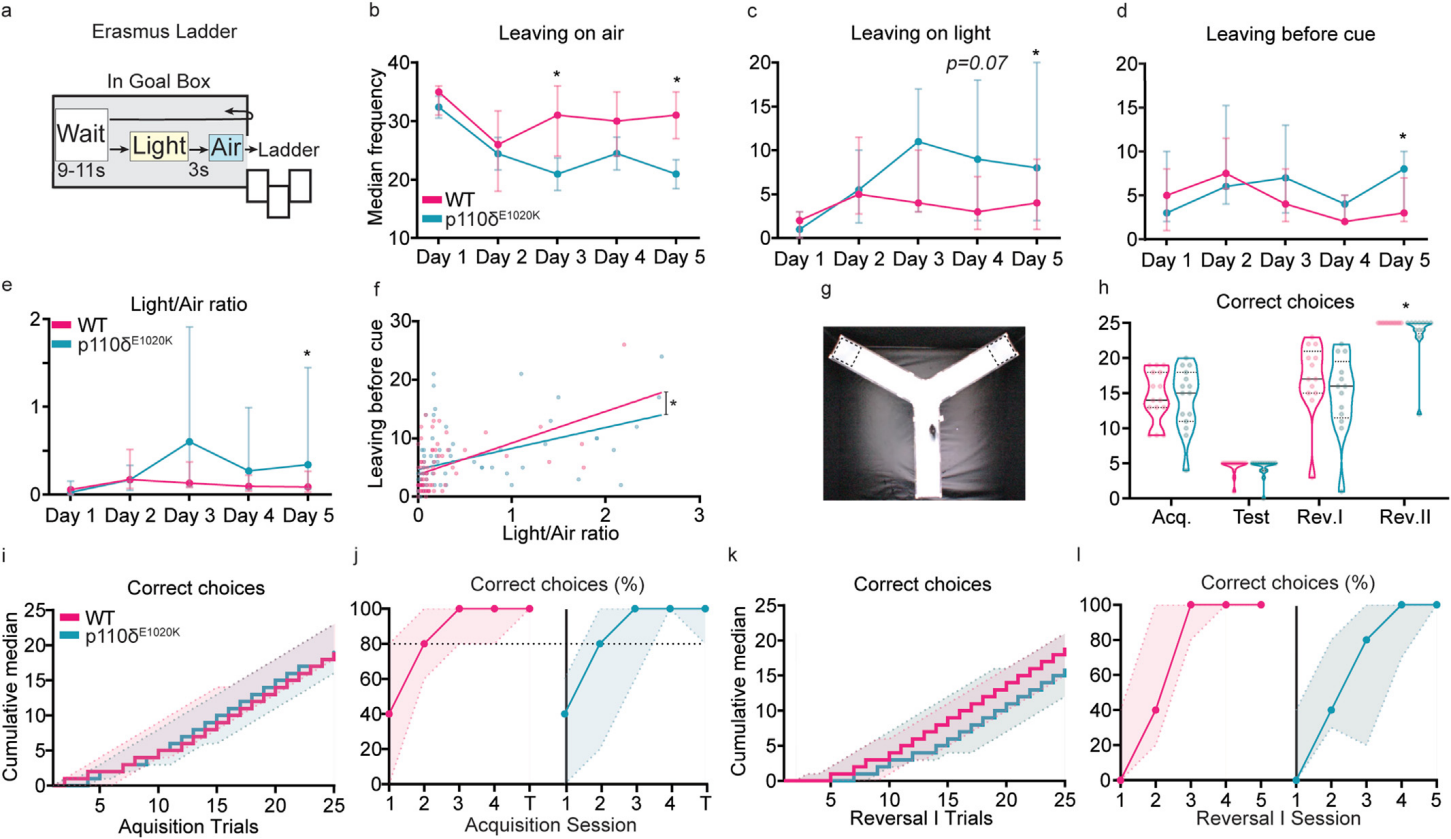
**Stimulus-dependent ladder exit and reversal learning are mildly affected in p110δ**^**E1020K**^**mice**. a) Schematic of the Erasmus ladder goal-box with the time intervals between stimuli. b-d) Number of times individual mice left the goal-box with the air stimulus (b), the light stimulus (c), or before light cue presentation (d). e) ratio between light and air exits. f) Best-fit regression model between the data points used to plot (d) and (e). g) Picture of the Y-Maze, with dashed squares representing the possible locations for the hidden platform, either on the right or left arm of the apparatus. h) Total number of correct arm choices for both genotypes, during each phase of the Y-Maze (data presented as median with interquartile range). i) Step function with the cumulative median and interquartile range for the number of correct arm choices during all acquisition and test trials. j) Percentage of correct arm choices for each genotype over the four days of acquisition and the day of test (data presented as median with interquartile range) k-l) Similar to (i) and (j) but for the reversal I phase. 2-tailed Mann-Whitney, except for f). ∗p ​≤ ​0.05, n ​= ​15 mice per genotype, except for reversal phases where n ​= ​13.

To investigate fine motor skills, mice were tested with the Erasmus ladder, a fully automated behavioural apparatus that allows detailed analysis and quantification of motor performance and learning in mice (Vinueza Veloz et al., 2015). p110δ^E1020K^ mice spent significantly less time crossing the ladder on the first two days of testing (Day 1: *U* ​= ​60, p ​= ​0.05; Day 2: *U* ​= ​40, p ​= ​0.01) (Fig. 3e). This was not prompted by a higher efficiency in crossing the ladder, as the percentage of missteps was similar for each day in both genotypes (main effect of genotype: *F* (Gruber and Bogunovic, 2020; Vries deet al, 2000) ​= ​1.786, p ​= ​0.19) (Supplementary Fig. 3c). We next analysed the locomotion pattern on the ladder (Fig. 3f). Although WT and p110δ^E1020K^ had identical percentages of backsteps (*F* (Gruber and Bogunovic, 2020; Vries deet al, 2000) ​= ​2.784, p ​= ​0.11) and longsteps (*F* (Gruber and Bogunovic, 2020; Vries deet al, 2000) ​= ​0.4735, p ​= ​0.50), p110δ^E1020K^ mice displayed a tendency to use a higher percentage of short steps (*F* (Gruber and Bogunovic, 2020; Vries deet al, 2000) ​= ​3.469, p ​= ​0.07; d ​= ​0.7) and used fewer jumps (F(1,28) ​= ​4.112, p ​= ​0.05; d ​= ​0.8) to cross the ladder (Fig. 3g). These pattern changes were independent of weight as this progressed similarly between groups (Supplementary Fig. 3d).

Together, these results indicate that PI3Kδ GOF mutation has no impact on gross motor function, but contributes to changes in fine locomotor skills that result in the adoption of a different locomotion strategy by mutant mice. This is in line with the findings in our patients, who do not present with gross motor function impairments either, but do present with fine-motor movement impairments.

### 3.5 p110δ^E1020K^*mice show altered patterns of repetitive behaviour independent of anxiety-like measures*

PIDs predispose patients to an increased prevalence of mood disorders (Kayan Ocakoglu et al., 2018), as does the presence of developmental delays (Cross et al., 2019; North et al., 2013). To further investigate anxiety-like behaviour in p110δ^E1020K^ mutants, we tested mice in the open-field (OF) and elevated-plus maze (EPM) tests.

In the OF, we found no evidence of increased anxiety-like behaviour in p110δ^E1020K^ mice, as both genotypes spent comparable time in the inner and outer areas of the arena (In: U ​= ​67, p ​= ​0.10; Out: U ​= ​103, p ​= ​0.71), as well as in the corners (U ​= ​111, p ​= ​97) (Fig. 4a and b, Supplementary Fig. 3b). There was also no effect of genotype in the EPM regarding the time spent on the different arms of the maze (F(1,28) ​= ​0.10, p ​= ​0.75) or the number of transitions between arms (F(1,28) ​= ​3.18, p ​= ​0.09) (Fig. 4c and d). These data indicate that p110δ^E1020K^ mice do not exhibit increased anxiety despite their immunological phenotype (Stark et al., 2018).

We next explored the presence of repetitive behaviours, a common comorbidity of neurodevelopmental delays (MacDuffie et al., 2020). Using the marble burying task (Supplementary Figs. 4a and b), we first measured the total area buried by each mouse and found this to be similar between genotypes (area buried: t(26) ​= ​0.43, p ​= ​0.67; number of buried marbles: t(26) ​= ​0.00, p ​> ​0.99) (Fig. 4e, Supplementary Fig. 4c). When the location of the buried marbles was mapped, we found that WT mice preferably buried marbles in the bottom right corner and centre, while p110δ^E1020K^ mice favoured areas close to the walls of the arena (Fig. 4f), indicating increased thigmotactic behaviour.

As the previous results suggested the presence of a distinct repetitive behaviour pattern, we further addressed this using the grooming assay. The total time spent grooming was similar between groups (t(28) ​= ​0.96, p ​= ​0.34) (Fig. 4g), as was the total number of grooming bouts (t(28) ​= ​1.65, p ​= ​0.11) (Fig. 4h) and latency to initiate grooming behaviour (t(28) ​= ​0.25, p ​= ​0.81) (Supplementary Fig. 4d). We found a tendency for the average time interval between grooming bouts to be smaller in p110δ^E1020K^ mice (t(28) ​= ​1.98, p ​= ​0.06; d ​= ​0.7) (Fig. 4i and j), further suggesting a difference in behaviour pattern between groups. Indeed, a significant interaction between genotype and short and long grooming bouts (genotype x type of bout: F(1,28) ​= ​5.31, p ​= ​0.03) revealed that p110δ^E1020K^ mice exhibited a higher prevalence of short bouts compared to WT, while the opposite was observed for the long bouts (short bouts: 13.29% in WT vs 19.46% in p110δ^E1020K^; long bouts: 86.71% in WT vs 80.54% in p110δ^E1020K^) (Supplementary Fig. 4e). Furthermore, when the number of grooming bouts was analysed over time, a tendency for increased bout number over time was seen in p110δ^E1020K^ mice towards the end of the assay (genotype x time: F(9,251) ​= ​2.15, p ​= ​0.03) (Fig. 4k, bottom curve). A significant time ​× ​genotype interaction was also found for the 3 ​min-binned time spent grooming (F(9,273) ​= ​3.51, p ​= ​0.0004) (Fig. 4k, top curve), further supporting the presence of an altered grooming pattern in p110δ^E1020K^ mice. Taken together, these data indicate that, despite the absence of increased anxiety-like behaviour, p110δ^E1020K^ mice present with subtle alterations in the pattern of repetitive behaviour.

### 3.6 p110δ^E1020K^ mice exhibit changes in associative response

Given the cognitive impairments and learning difficulties presented by some APDS patients (Jamee et al., 2019; Elkaim et al., 2016), including P1, we investigated associative and spatial learning in the p110δ^E1020K^ mice.

First, we quantified learning using the Erasmus ladder. During the Erasmus ladder task, two stimuli are presented. Initially, a light turns on inside the goal box. Next, an air stream encourages the mouse to enter the ladder (Vinueza Veloz et al., 2015) (Fig. 5a). Considering the exit frequency for each stimulus, we found that both genotypes responded similarly to stimuli in the first sessions of the task. For sessions 3 and 5, p110δ^E1020K^ mice left the goal box less frequently with the air stimulus than WT (session 3: U ​= ​61, p ​= ​0.03; session 5: U ​= ​55.5, p ​= ​0.02) (Fig. 5b), while increasing box exits after light presentation in later sessions (session 4: U ​= ​63.5, p ​= ​0.07; session 5: U ​= ​63.5, p ​= ​0.04) (Fig. 5c,e). Increased light exit frequency could be representative of increased readiness or impulsivity to leave the box, interrupting the pre-stimulus waiting period. From testing days 1–4, both genotypes left the box before cue presentation with similar frequencies (Fig. 5d). On day 5, this frequency was increased in p110δ^E1020K^ mice (session 5: U ​= ​60.5, p ​= ​0.05). As expected, there was a positive association between leaving before cue and the light/air exit ratio (WT: ⍴ ​= ​0.49, p ​< ​0.0001; p110δ^E1020K^: ⍴ ​= ​0.63, p ​< ​0.0001). Least squares fitting demonstrated that the response of the two genotypes to the stimuli was significantly different (F(2,139) ​= ​3.906, p ​= ​0.02; WT: y ​= ​4.9x+3.8; p110δ^E1020K^: y ​= ​2.3x+4.5) (Fig. 5f).

To further explore learning behaviour, we used the water Y-maze (Fig. 5g), a test often used to study repetitive behaviour and cognitive flexibility in ASD-mouse models (Tsai et al., 2012; Stoodley et al., 2017; Badura et al., 2018). Similar to the previous OF and Rotarod results, we found no evidence of motor dysfunction, with both genotypes swimming similar distances and at comparable speeds during the habituation phase (distance: U ​= ​67, p ​= ​0.22; speed: U ​= ​77, p ​= ​0.23) (Supplementary Figs. 4f and g). During the acquisition and test phases, both WT and p110δ^E1020K^ mice learned the platform location, and there was no difference in the number of correct arm choices made by each genotype (Fig. 5i and j). When the location of the platform was reversed, p110δ^E1020K^ mice presented with a lower cumulative median of correct choices per trial, taking longer to perform the task correctly (Fig. 5k). No significant differences were found in the total number of correct choices per session (Fig. 5l). Similar results were obtained regarding the reversal II phase (Supplementary Figs. 4h and i). However, in this phase, errors in platform arm choice were only performed by p110δ^E1020K^ mice (U ​= ​45.5, p ​= ​0.01). Taken together, these results indicate that p110δ^E1020K^ mice present with mild deficits in paired-stimulus learning and reversal learning.

### 3.7 p110δ^E1020K^ mice display intact social interaction behaviour

Atypical development of social skills and interactions is a common component of neuropsychiatric conditions, particularly of those with ASD comorbidity (Siegel and Smith, 2010; Brown et al., 2020). Therefore, we sought to evaluate the performance of p110δ^E1020K^ mice in a social interaction paradigm (Yang et al., 2011).

**Fig. 6:**
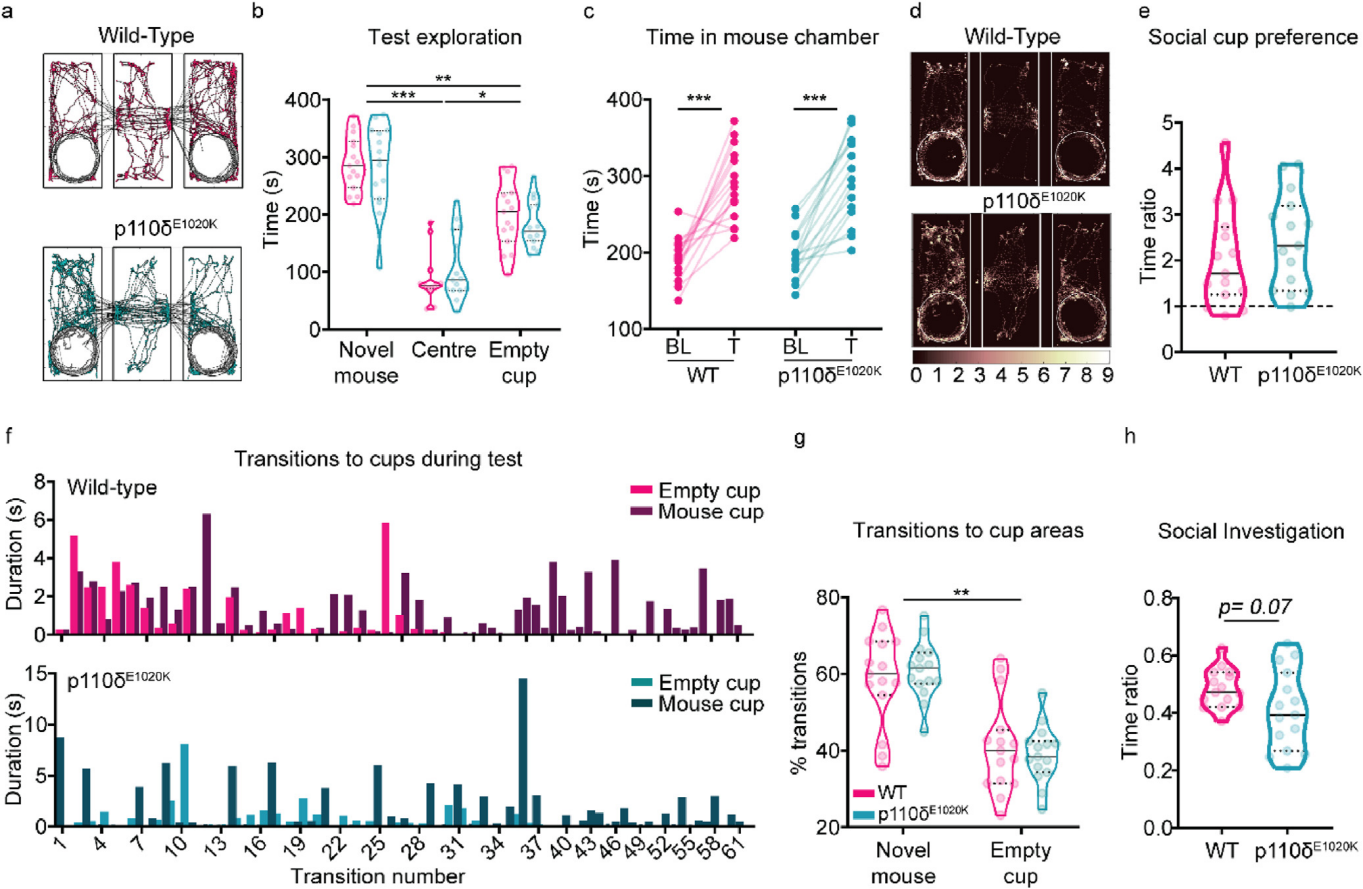
**Social behaviour is largely preserved in p110δ**^**E1020K**^**mice**. a) Example of automatically tracked body positions during test phase (novel mouse on the left). b) Total time individual mice spent in each chamber of the apparatus during test phase (mixed effects model; data presented as median with interquartile range). c) Before and after plot of the total time each individual mouse spent on the novel mouse chamber during baseline (BL) and test (T) (2-way repeated-measures ANOVA). d) Body position heatmap depicting position frequency per 2.5 ​mm bins (novel mouse on the left). e) Median and quartiles with the ratio between the time each individual mouse spent near the social cup over the time it spent near the empty cup (2-tailed *t*-test). f) Representative plot with the duration, in seconds, of each transition into the empty (light bars) or novel mouse (dark bars) cup area. g) Median and quartiles with the percentage of transitions each individual mouse made to the novel mouse or empty cup (2-way repeated-measures ANOVA); h) Median and quartiles of the ratio between the time individual mice spent exploring the novel mouse cup over the time spent in the whole novel mouse chamber (2-tailed *t*-test). ∗p ​≤ ​0.05, ∗∗p ​≤ ​0.01, ∗∗∗p ​≤ ​0.001. n ​= ​15 mice per genotype.

During baseline exploration of the three-chamber apparatus, when no social stimulus was presented, both genotypes displayed a similar ambulatory behaviour across all chambers (F(1,28) ​= ​0.5376, p ​= ​0.47) (Supplementary Figs. 5a and c). p110δ^E1020K^ mice displayed slightly altered exploratory behaviour, with a tendency for centre crossing avoidance (genotype x chamber: F(2,55) ​= ​2.988, p ​= ​0.06; WT mean centre transitions ​= ​46.57 vs p110δ^E1020K^ mean centre transitions ​= ​38.40) (Supplementary Fig. 5d). During the test phase, a novel mouse was introduced to the arena (Fig. 6a). Both WT and p110δ^E1020K^ mice spent more time in the chamber where the novel mouse was located (main effect of chamber: F(1.911,79.30) ​= ​87.71, p ​< ​0.0001; main effect of genotype: F(1,83) ​= ​0.0006, p ​= ​0.98) (Fig. 6b), increasing the time spent in this chamber compared to their correspondent baseline values (main effect of phase: F(1,28) ​= ​98.74, p ​< ​0.0001; main effect of genotype: F(1,28) ​= ​0.2441, p ​= ​0.63) (Fig. 6c). Similar to what was found for the baseline exploration period, the avoidance of central area crossings in p110δ^E1020K^ mice persisted in the test phase (genotype x chamber: F(2,54) ​= ​5.423, p ​= ​0.01; WT mean centre transitions ​= ​27.21 vs p110δ^E1020K^ mean centre transitions ​= ​23.64) (Supplementary Fig. 5e).

Focusing on the region of interest defined around the empty cup and the cup with the novel mouse, both genotypes demonstrated a comparable preference for interacting with the cup where the social stimulus was located (t(28) ​= ​0.99, p ​= ​0.33), spending approximately twice the time exploring this cup compared to the empty cup (Fig. 6d and e). This preference for social cup exploration was also accompanied by an increased number of transitions into the novel mouse cup area (main effect of cup: F(1,28) ​= ​29.04, p ​< ​0.0001) (Fig. 6f and g). For both genotypes, the time spent exploring the novel social stimulus progressively decreased over the course of the task (main effect of time: F(1.328,118.8) ​= ​5.714, p ​= ​0.0002; main effect of genotype: F(1,28) ​= ​0.3303, p ​= ​0.57) (Supplementary Fig. 5b). Finally, when social investigation preference was analysed, p110δ^E1020K^ mice exhibited a tendency to spend a lower proportion of their time in the novel mouse chamber in the proximity of the cup, although this did not reach the statistical significance threshold (t(28) ​= ​1.877, p ​= ​0.07; d ​= ​0.7) (Fig. 6h). Altogether, these data indicate that, despite a slight centre avoidance phenotype, p110δ^E1020K^ mice prefer the social stimulus over the asocial one, exhibiting an unaffected social phenotype.

### 3.8 Motor, learning and repetitive behaviours best discriminate WT and p110δ^E1020K^ mouse populations

**Fig. 7:**
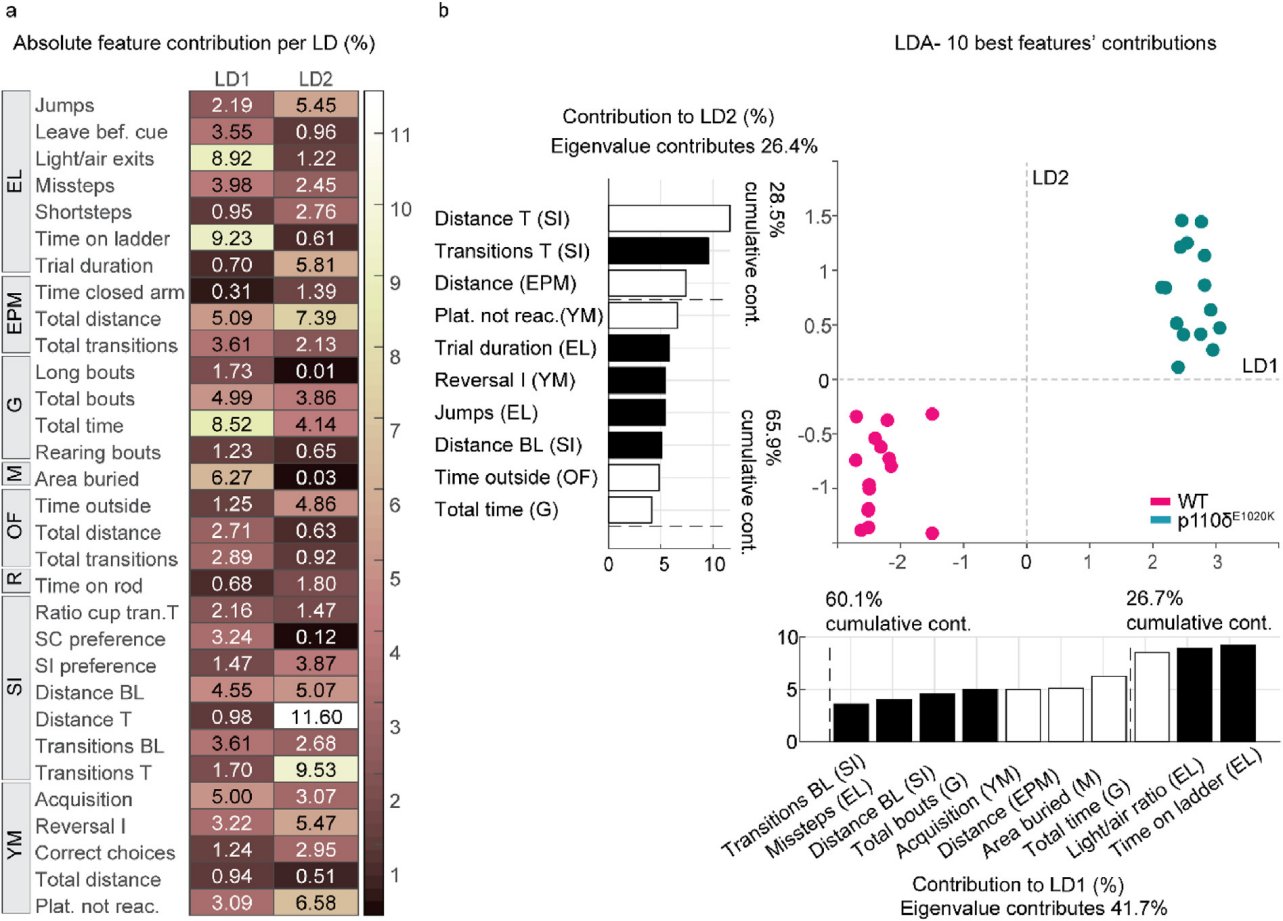
**LD1 and LD2 features separate WT from p110δ**^**E1020K**^**mice**. a) Absolute contribution of each behavioural variable, in percentage, to linear discriminants 1 and 2, resulting from the LDA. b) LDA plot featuring the 10 best contributors to LD1 and LD2. Negative and positive contributions are represented by black and white bars, respectively. Each dot represents one mouse, with pink dots representing WT mice and green dots p110δ^E1020K^ mice. n ​= ​15 mice per genotype. EL, Erasmus ladder, EPM, elevated-plus maze, G, grooming, M, marble burying, OF, open-field, R, rotarod, SI, social interaction, YM, water y-maze. (For interpretation of the references to color in this figure legend, the reader is referred to the Web version of this article.)

The analysis of independent readouts for each behaviour revealed a number of discrete changes in the behavioural pattern of p110δ^E1020K^ mice. Nonetheless, behaviour is a dynamic process where small stereotyped modules are often grouped or combined into larger representations that underlie each individual's phenotype (Datta et al., 2019; Berman, 2018). To better understand the most important contributors to the phenotype of p110δ^E1020K^ mice, we performed linear discriminant analysis (LDA) on all behavioural variables measured (Machado et al., 2020; Forkosh et al., 2019). This type of analysis allows for encompassing individual differences across individuals and captures stable traits best separating the genotypes across many tests (Forkosh et al., 2019). We then selected the first two dimensions, LD1 and LD2 (Fig. 7a), and plotted the 10 best contributing components of each discriminant, as these are the variables that give the most information on group separation (Fig. 7b).

LDA of the behavioural data classified individual points into 2 non-overlapping classes, identifying the two genotypes. The 2 best LDs represent 68.1% of data variation, with LD1, which explains 41.7% of total data variation, creating a maximal separation between classes. Focusing on the greatest weights, motor and learning related variables (*time on ladder* and *light to air ratio*, respectively) contribute the most for group classification. The third feature, *total grooming time*, with an absolute contribution of 8.5%, indicates that additional group separation is achieved by the inclusion of repetitive behaviours in this discriminant. Further separation of the data along the vertical axis is provided by LD2, albeit with lower contributions (26.4%). This discriminant represents parameters predominantly influenced by locomotion-derived features. These include total distance travelled and transitions made during the test phase of the SI, and total distance travelled during the EPM. Altogether, these results indicate that LDA compiles and captures behavioural alterations in locomotor performance, learning and repetitive behaviours between WT and p110δ^E1020K^ mice, supporting the previously identified univariate analysis findings.

## 4 Discussion

The study of the immune system in the regulation of neurodevelopment and in shaping subsequent behaviour is a rapidly emerging field, involving crosstalk in immunoneuropsychiatry and new integrative therapeutic approaches (Deverman and Patterson, 2009; Cowan and Petri, 2018; Pape et al., 2019). In this work, we investigated neurologically-relevant behavioural features in APDS, a rare PID, using both patient data and a murine model. To our knowledge, this is the first study of APDS which specifically focuses on its behavioural component.

APDS patients exhibited changes in visuomotor responses, with P1 presenting with motor integration deficits, while both patients displayed decreased memory recall capacity. P1 was also formally diagnosed with ASD, strengthening previous more general reports describing neurodevelopmental delay as a possible comorbidity of APDS patients (Coulter et al., 2017; Elkaim et al., 2016; Wang et al., 2018; Ahmed et al., 2020). In the p110δ^E1020K^ murine model, we detected a more subtle phenotype. GOF mice presented with altered patterns of locomotion and repetitive behaviours, features reminiscent of symptoms found in individuals with ASD (MacDuffie et al., 2020; Ming et al., 2007).

While our data suggested a role of *PIK3CD* GOF on behaviour, the precise function of PI3Kδ in the brain remains elusive. In mice, p110δ has been found in brain and spinal cord, and proposed to have a role in neuronal morphology (Nieuwenhuis et al., 2020; Eickholt et al., 2007; Hood et al., 2020; Schmidt et al., 2014). Although the expression pattern of human *PIK3CD* follows a similar distribution as in mice (Allen Human Brain Atlas (2010)), reports of its non-immunological functions are scarce, with only a few studies implicating this isoform in schizophrenia and autism (Hood et al., 2019; Poopal et al., 2016; Law et al., 2012). The presence of some behavioural deficits in adult mice combined with the low PI3Kδ expression in the brain, suggests that this isoform might have a predominant function during brain development rather than adulthood. Consistent with this hypothesis, recent studies found more PIK3CD transcripts in human foetal brain than in adult samples (Hood et al., 2019), and distributed expression of *PIK3CD* in developing mouse brain (La Manno et al., 2020).

Despite its presence in the brain, PI3Kδ is predominantly expressed in leukocytes. This supports a putative modulatory role of the immune system on behaviour. Accordingly, a number of studies has now suggested a link between neurodevelopmental and immunological dysregulation. For example, in rodents, externally triggering a maternal immune response during pregnancy induces behavioural alterations in adult offspring. These include reduced cognitive flexibility and decreased social exploration, traits of an ASD-like phenotype (Shi et al., 2003; Haida et al., 2019; Amodeo et al., 2019). In humans, increased odds of neonatal infections were reported for children with ASD (Sabourin et al., 2019). Additionally, viral or bacterial infections during pregnancy were associated with an increased likelihood of ASD diagnosis (Lee et al., 2015; Atladóttir et al., 2010), whereas increased ASD symptom severity was found in children with a maternal history of chronic immune activation (Patel et al., 2018). In APDS, family history of immunodeficiency is also estimated in 39% of patients (Jamee et al., 2019), thus suggesting that PID could be important predictors of behaviour dysfunction. Strengthening the hypothesis of an *immunological-behavioural phenotype* relationship is the presentation of the described patients, with P1 displaying both increased immunodeficiency and visuomotor impairments when compared to P2, who presents with a milder phenotype (Yildirim et al., 2018; Subbarayan et al., 2011).

The interplay between the immune system and the brain is a current topic of rapid scientific discovery (Meltzer and Water, 2017; Nazeen et al., 2016). Here, we show that a heterozygous mouse model of APDS displays mild behavioural alterations in addition to its immunological phenotype. ADPS patients showed high levels of heterogeneity when it came to behavioural and immunological symptoms. However, both P1 and P2 presented with sensorimotor deficits, a feature captured by the mouse model. Notably, the severity of the immunodeficiency symptoms between P1 and P2 was reflected in the performance during the visuomotor tests. This is of interest to the APDS community, because such tests have previously been shown to accurately capture the features of early stage Alzheimer's disease (Tippett and Sergio, 2006), correlate with cognitive impairments in Parkinson's disease (Inzelberg et al., 2008), and serve as a tool to monitor the progression of both conditions (de Boer et al., 2016). Further, due to their non-invasive nature, such tests are suitable to use even in very young children (Pel et al., 2016). In the future, we aim to further explore the correlation between immune system impairments, behavioural deficits and the outcome of the visuomotor deficits, on a larger APDS patient cohort, assessing the potential benefits of including this type of test batteries in the diagnostic pathway.

In addition to reinforcing the need for a multidisciplinary team assessing APDS patients, this study highlights the importance of increased monitoring of immunodeficient patients for the presence of neuropsychiatric comorbidities and describes a set of non-invasive tools that allow for such assessment. Additional studies on the function of PI3Kδ in the brain will be fundamental to understand its specific role in neurodevelopment and deepen our knowledge of the interactions between immunological burden and neuropsychiatric load.

## Author contributions

IS, AB, VASHD and JJMP designed and supervised the study. AB, KO, VASHD, JJMP provided resources and acquired funding. VASHD, ORM, SMA and NJMB identified patients and performed clinical diagnosis. IIF, JJMP and AB performed the visuomotor experiments. IS and LW performed mouse experiments. FMPK and HI performed in vitro experiments. IS, IIF, LW and CVDZ performed data analysis. IS performed statistical analysis and prepared figures. IS, AB, VASHD and JJMP wrote the first draft. All authors edited the manuscript.

IS and ORM share first authorship. ORM and VASHD provided the clinical characterisation of P1 and P2, whereas IS performed all mouse experiments and coordinated visuomotor data collection and analysis. Because IS drafted the paper, they are listed first.

## Declaration of competing interest

We declare that one of the authors (PMH) has received grants and research support from Takeda, CSL Behring, Abbvie, Lamepro, Novartis Nederland, and honoraria or consultation fees from UCB Pharma. All the other authors have no conflict of interest to declare.

## References

[bib1] Abolhassani H. (2018). Clinical, immunologic, and genetic spectrum of 696 patients with combined immunodeficiency. J. Allergy Clin. Immunol..

[bib2] Ahmed A.A., Shahaway El, A A., Hussien S.A. (2020). Activated PI3K-delta syndrome in an Egyptian pediatric cohort with primary immune deficiency. Allergol. Immunopathol..

[bib3] Amaya-Uribe L., Rojas M., Azizi G., Anaya J.-M., Gershwin M.E. (2019). Primary immunodeficiency and autoimmunity: a comprehensive review. J. Autoimmun..

[bib4] Amodeo D.A. (2019). Maternal immune activation impairs cognitive flexibility and alters transcription in frontal cortex. Neurobiol. Dis..

[bib5] Angulo I. (2013). Phosphoinositide 3-kinase δ gene mutation predisposes to respiratory infection and airway damage. Science.

[bib6] Atladóttir H.O. (2010). Maternal infection requiring hospitalization during pregnancy and autism spectrum disorders. J. Autism Dev. Disord..

[bib7] Badura A. (2018). Normal cognitive and social development require posterior cerebellar activity. Elife.

[bib8] Baron-Cohen S., Wheelwright S., Skinner R., Martin J., Clubley E. (2001). The autism-spectrum quotient (AQ): evidence from Asperger syndrome/high-functioning autism, males and females, scientists and mathematicians. J. Autism Dev. Disord..

[bib9] Ben-Shaul Y. (2017). OptiMouse: a comprehensive open source program for reliable detection and analysis of mouse body and nose positions. BMC Biol..

[bib10] Berman G.J. (2018). Measuring behavior across scales. BMC Biol..

[bib11] Bethune C. (2019). British Society for Immunology/United Kingdom Primary Immunodeficiency Network consensus statement on managing non-infectious complications of common variable immunodeficiency disorders. Clin. Exp. Immunol..

[bib12] Bishop J.C., Pangelinan M. (2018). Motor skills intervention research of children with disabilities. Res. Dev. Disabil..

[bib13] Brown K.A., Parikh S., Patel D.R. (2020). Understanding basic concepts of developmental diagnosis in children. Transl. Pediatr..

[bib15] Chantry D. (1997). p110delta, a novel phosphatidylinositol 3-kinase catalytic subunit that associates with p85 and is expressed predominantly in leukocytes. J. Biol. Chem..

[bib16] Choi B., Leech K.A., Tager-Flusberg H., Nelson C.A. (2018). Development of fine motor skills is associated with expressive language outcomes in infants at high and low risk for autism spectrum disorder. J. Neurodev. Disord..

[bib17] Clayton E. (2002). A crucial role for the p110delta subunit of phosphatidylinositol 3-kinase in B cell development and activation. J. Exp. Med..

[bib19] Costa-Mattioli M., Monteggia L.M. (2013). mTOR complexes in neurodevelopmental and neuropsychiatric disorders. Nat. Neurosci..

[bib20] Coulter T.I. (2017). Clinical spectrum and features of activated phosphoinositide 3-kinase δ syndrome: a large patient cohort study. J. Allergy Clin. Immunol..

[bib21] Cowan M., Petri W.A. (2018). Microglia: immune regulators of neurodevelopment. Front. Immunol..

[bib22] Cross A.J., Goharpey N., Laycock R., Crewther S.G. (2019). Anxiety as a common biomarker for school children with additional health and developmental needs irrespective of diagnosis. Front. Psychol..

[bib23] Datta S.R., Anderson D.J., Branson K., Perona P., Leifer A. (2019). Computational neuroethology: a call to action. Neuron.

[bib24] de Boer C., van der Steen J., Mattace-Raso F., Boon A.J.W., Pel J.J.M. (2016). The effect of neurodegeneration on visuomotor behavior in alzheimer's disease and Parkinson's disease. Mot. Control.

[bib25] Del Bianco T. (2020). Temporal profiles of social attention are different across development in autistic and neurotypical people. Biol Psychiatry Cogn Neurosci Neuroimaging.

[bib26] Deverman B.E., Patterson P.H. (2009). Cytokines and CNS development. Neuron.

[bib27] Dornan G.L. (2017). Conformational disruption of PI3Kδ regulation by immunodeficiency mutations in PIK3CD and PIK3R1. Proc. Natl. Acad. Sci. U. S. A.

[bib28] Duda R.O., Hart P.E., Stork D.G. (1973). Pattern classification and scene analysis. 3, (Wiley New York.

[bib29] Eickholt B.J. (2007). Control of axonal growth and regeneration of sensory neurons by the p110delta PI 3-kinase. PLoS One.

[bib30] Elkaim E. (2016). Clinical and immunologic phenotype associated with activated phosphoinositide 3-kinase δ syndrome 2: a cohort study. J. Allergy Clin. Immunol..

[bib31] Everling S., Fischer B. (1998). The antisaccade: a review of basic research and clinical studies. Neuropsychologia.

[bib32] Forkosh O. (2019). Identity domains capture individual differences from across the behavioral repertoire. Nat. Neurosci..

[bib33] Friard O., Gamba M. (2016). Boris : a free, versatile open-source event-logging software for video/audio coding and live observations. Methods Ecol. Evol..

[bib34] Gomez M.J. (1990). Comparison of methods for outlier detection and their effects on the classification results for a particular data base. Anal. Chim. Acta.

[bib35] Gruber C., Bogunovic D. (2020). Incomplete penetrance in primary immunodeficiency: a skeleton in the closet. Hum. Genet..

[bib36] Haida O. (2019). Sex-dependent behavioral deficits and neuropathology in a maternal immune activation model of autism. Transl. Psychiatry.

[bib37] Hood V.L., Berger R., Freedman R., Law A.J. (2019). Transcription of PIK3CD in human brain and schizophrenia: regulation by proinflammatory cytokines. Hum. Mol. Genet..

[bib38] Hood V.L., Paterson C., Law A.J. (2020). PI3Kinase-p110δ overexpression impairs dendritic morphogenesis and increases dendritic spine density. Front. Mol. Neurosci..

[bib39] Inzelberg R., Schechtman E., Hocherman S. (2008). Visuo-motor coordination deficits and motor impairments in Parkinson's disease. PLoS One.

[bib40] Jamee M. (2019). Clinical, Immunological, and Genetic Features in Patients with Activated PI3Kδ Syndrome (APDS): a Systematic Review. Clin. Rev. Allergy Immunol.

[bib41] Jossin Y., Goffinet A.M. (2007). Reelin signals through phosphatidylinositol 3-kinase and Akt to control cortical development and through mTor to regulate dendritic growth. Mol. Cell Biol..

[bib42] Kang S.S., Dionisio D.P., Sponheim S.R. (2011). Abnormal mechanisms of antisaccade generation in schizophrenia patients and unaffected biological relatives of schizophrenia patients. Psychophysiology.

[bib44] Kayan Ocakoglu B., Karaca N.E., Ocakoglu F.T., Erermis S. (2018). Psychological burden of pediatric primary immunodeficiency. Pediatr. Int..

[bib45] La Manno G. (2020).

[bib46] Landau D.P., Binder K. (2005).

[bib47] Law A.J. (2012). Neuregulin 1-ErbB4-PI3K signaling in schizophrenia and phosphoinositide 3-kinase-p110δ inhibition as a potential therapeutic strategy. Proc. Natl. Acad. Sci. U. S. A.

[bib48] Lee B.K. (2015). Maternal hospitalization with infection during pregnancy and risk of autism spectrum disorders. Brain Behav. Immun.

[bib49] MacDuffie K.E. (2020). Sleep problems and trajectories of restricted and repetitive behaviors in children with neurodevelopmental disabilities. J. Autism Dev. Disord..

[bib50] Machado A.S., Marques H.G., Duarte D.F., Darmohray D.M., Carey M.R. (2020). Shared and specific signatures of locomotor ataxia in mutant mice. Neuroscience.

[bib51] Meltzer A., Water Van de (2017). J. The Role of the Immune System in Autism Spectrum Disorder. Neuropsychopharmacology.

[bib52] Meyniel C., Rivaud-Péchoux S., Damier P., Gaymard B. (2005). Saccade impairments in patients with fronto-temporal dementia. J. Neurol. Neurosurg. Psychiatry.

[bib53] Ming X., Brimacombe M., Wagner G.C. (2007). Prevalence of motor impairment in autism spectrum disorders. Brain Dev..

[bib54] Mostofsky S.H. (2009). Decreased connectivity and cerebellar activity in autism during motor task performance. Brain.

[bib55] Mous S.E., Jiang A., Agrawal A., Constantino J.N. (2017). Attention and motor deficits index non-specific background liabilities that predict autism recurrence in siblings. J. Neurodev. Disord..

[bib57] Muilwijk D., Verheij S., Pel J.J., Boon A.J., van der Steen J. (2013). Changes in Timing and kinematics of goal directed eye-hand movements in early-stage Parkinson's disease. Transl. Neurodegener..

[bib58] Munoz D.P., Everling S. (2004). Look away: the anti-saccade task and the voluntary control of eye movement. Nat. Rev. Neurosci..

[bib59] Müri R.M., Iba-Zizen M.T., Derosier C., Cabanis E.A., Pierrot-Deseilligny C. (1996). Location of the human posterior eye field with functional magnetic resonance imaging. J. Neurol. Neurosurg. Psychiatry.

[bib60] Nazeen S., Palmer N.P., Berger B., Kohane I.S. (2016). Integrative analysis of genetic data sets reveals a shared innate immune component in autism spectrum disorder and its co-morbidities. Genome Biol..

[bib61] Ng M.K., Liao L.-Z., Zhang L. (2011). On sparse linear discriminant analysis algorithm for high-dimensional data classification. Numer. Lin. Algebra Appl..

[bib62] Nieman D. (2007). Antisaccade task performance in patients at ultra high risk for developing psychosis. Schizophr. Res..

[bib63] Nieuwenhuis B. (2020). PI 3-kinase delta enhances axonal PIP3 to support axon regeneration in the adult CNS. EMBO Mol. Med. e11674.

[bib64] North C.R., Wild T.C., Zwaigenbaum L., Colman I. (2013). Early neurodevelopment and self-reported adolescent symptoms of depression and anxiety in a National Canadian Cohort Study. PLoS One.

[bib65] Nunes-Santos C.J., Uzel G., Rosenzweig S.D. (2019). PI3K pathway defects leading to immunodeficiency and immune dysregulation. J. Allergy Clin. Immunol..

[bib66] Odnoletkova I. (2018). The burden of common variable immunodeficiency disorders: a retrospective analysis of the European Society for Immunodeficiency (ESID) registry data. Orphanet J. Rare Dis..

[bib68] Pape K., Tamouza R., Leboyer M., Zipp F. (2019). Immunoneuropsychiatry - novel perspectives on brain disorders. Nat. Rev. Neurol..

[bib69] Patel S. (2018). Social impairments in autism spectrum disorder are related to maternal immune history profile. Mol. Psychiatr..

[bib70] Pel J.J.M. (2016). Early identification of cerebral visual impairments in infants born extremely preterm. Dev. Med. Child Neurol..

[bib71] Pierrot-Deseilligny C., Milea D., Müri R.M. (2004). Eye movement control by the cerebral cortex. Curr. Opin. Neurol..

[bib72] Piu P. (2019). The cerebellum improves the precision of antisaccades by a latency-duration trade-off. Prog. Brain Res..

[bib73] Poopal A.C., Schroeder L.M., Horn P.S., Bassell G.J., Gross C. (2016). Increased expression of the PI3K catalytic subunit p110δ underlies elevated S6 phosphorylation and protein synthesis in an individual with autism from a multiplex family. Mol. Autism..

[bib75] Prut L., Belzung C. (2003). The open field as a paradigm to measure the effects of drugs on anxiety-like behaviors: a review. Eur. J. Pharmacol..

[bib77] Reith R.M., Way S., McKenna J., Haines K., Gambello M.J. (2011). Loss of the tuberous sclerosis complex protein tuberin causes Purkinje cell degeneration. Neurobiol. Dis..

[bib79] Sabourin K.R. (2019). Infections in children with autism spectrum disorder: study to explore early development (SEED). Autism Res..

[bib80] Schindelin J. (2012). Fiji: an open-source platform for biological-image analysis. Nat. Methods.

[bib81] Schmidt J.T., Mariconda L., Morillo F., Apraku E. (2014). A role for the polarity complex and PI3 kinase in branch formation within retinotectal arbors of zebrafish. Dev. Neurobiol..

[bib83] Shi L., Fatemi S.H., Sidwell R.W., Patterson P.H. (2003). Maternal influenza infection causes marked behavioral and pharmacological changes in the offspring. J. Neurosci..

[bib84] Siegel M.S., Smith W.E. (2010). Psychiatric features in children with genetic syndromes: toward functional phenotypes. Child Adolesc. Psychiatr. Clin. N. Am..

[bib85] Sonzogni M. (2018). A behavioral test battery for mouse models of Angelman syndrome: a powerful tool for testing drugs and novel Ube3a mutants. Mol. Autism..

[bib86] Stark A.-K. (2018). PI3Kδ hyper-activation promotes development of B cells that exacerbate Streptococcus pneumoniae infection in an antibody-independent manner. Nat. Commun..

[bib88] Stoodley C.J. (2017). Altered cerebellar connectivity in autism and cerebellar-mediated rescue of autism-related behaviors in mice. Nat. Neurosci..

[bib89] Subbarayan A. (2011). Clinical features that identify children with primary immunodeficiency diseases. Pediatrics.

[bib91] Tee A.R., Sampson J.R., Pal D.K., Bateman J.M. (2016). The role of mTOR signalling in neurogenesis, insights from tuberous sclerosis complex. Semin. Cell Dev. Biol..

[bib92] Thauland T.J., Pellerin L., Ohgami R.S., Bacchetta R., Butte M.J. (2019). Case study: mechanism for increased follicular helper T cell development in activated PI3K delta syndrome. Front. Immunol..

[bib93] Thaventhiran J.E.D. (2020). Whole-genome sequencing of a sporadic primary immunodeficiency cohort. Nature.

[bib94] Tippett W.J., Sergio L.E. (2006). Visuomotor integration is impaired in early stage Alzheimer's disease. Brain Res..

[bib95] Tsai P.T. (2012). Autistic-like behaviour and cerebellar dysfunction in Purkinje cell Tsc1 mutant mice. Nature.

[bib97] van den Boom B.J.G., Pavlidi P., Wolf C.J.H., Mooij A.H., Willuhn I. (2017). Automated classification of self-grooming in mice using open-source software. J. Neurosci. Methods.

[bib98] Van Der Werf J., Buchholz V.N., Jensen O., Medendorp W.P. (2009). Neuronal synchronization in human parietal cortex during saccade planning. Behav. Brain Res..

[bib99] Vinueza Veloz M.F. (2015). Cerebellar control of gait and interlimb coordination. Brain Struct. Funct.

[bib100] Vries de E. (2000). Immulogie in de medische praktijk. XXXIV. Diagnostiek bij vermoeden van een afweerstoornis: inleiding.

[bib101] Waldthaler J., Tsitsi P., Svenningsson P. (2019). Vertical saccades and antisaccades: complementary markers for motor and cognitive impairment in Parkinson's disease. NPJ Parkinsons Dis.

[bib102] Wang Y. (2018). Report of a Chinese cohort with activated phosphoinositide 3-kinase δ syndrome. J. Clin. Immunol..

[bib103] Wentink M. (2017). Genetic defects in PI3Kδ affect B-cell differentiation and maturation leading to hypogammaglobulineamia and recurrent infections. Clin. Immunol..

[bib104] Yang M., Silverman J.L., Crawley J.N. (2011). Automated three-chambered social approach task for mice. Curr. Protoc. Neurosci.

[bib105] Yildirim M. (2018). Neurologic involvement in primary immunodeficiency disorders. J. Child Neurol..

