# Supplementary material for: Activated PI3Kδ syndrome, an immunodeficiency disorder, leads to sensorimotor deficits recapitulated in a murine model
Source: Brain Behav Immun Health. 2021 Oct 19;18:100377. doi: 10.1016/j.bbih.2021.100377 (PMC8579111; doi:10.1016/j.bbih.2021.100377)
Supplement: Multimedia component 1 [file mmc1.docx]

**Activated PI3Kδ syndrome, a primary immunodeficiency disorder, leads to sensorimotor deficits recapitulated in a murine model**

Ines Serra^1*^, Olivia R. Manusama^2*^, Fabian M. P. Kaiser^2,3^, Izi Izumi Floriano^1^, Lucas Wahl^1^, Christian van der Zalm^1^, Hanna IJspeert^2^, P. Martin van Hagen^2,4^, Nico J.M. van Beveren^5^, Sandra M. Arend^6^, Klaus Okkenhaug^7^, Johan J.M. Pel^1^, Virgil A.S.H. Dalm^2,4,8^ and Aleksandra Badura^1^

^1^ Department of Neuroscience, Erasmus MC, Rotterdam, The Netherlands

^2^ Department of Immunology, Erasmus MC, Rotterdam, The Netherlands

^3^ Department of Pediatrics, Erasmus MC, Rotterdam, The Netherlands

^4^ Division of Clinical Immunology, Department of Internal Medicine, Erasmus MC, Rotterdam, The Netherlands

^5^ Department of Psychiatry, Erasmus MC, Rotterdam, The Netherlands

^6s^Department of Infectious Diseases, Leiden University Medical Center, Leiden, The Netherlands

^7^ Department of Pathology, University of Cambridge, Cambridge, United Kingdom

^8^ Academic Center for Rare Immunological Diseases (RIDC), Erasmus MC, Rotterdam, The Netherlands

**Correspondence**: Aleksandra Badura (a.badura@erasmusmc.nl)

Department of Neuroscience, Erasmus MC

Wytemaweg 80, 3015 CN Rotterdam

tel: 0031-(0)10-7043309

**Supplemental figures**

**
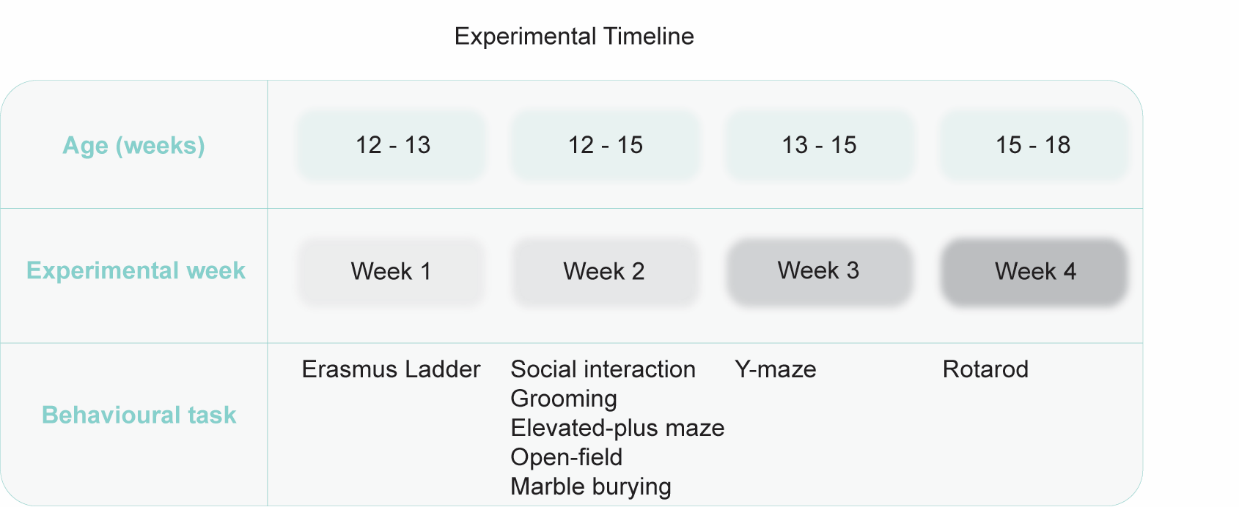
**

**Figure S1** Experimental timeline. WT and p110δ^E1020K^ mice were tested across four experimental weeks, in eight behavioural tasks. Behavioural experiments were initiated when mice were 11 to 13 weeks old and finished when mice were 15 to 18 weeks old. Brain tissue was collected at the end of experimental week 4


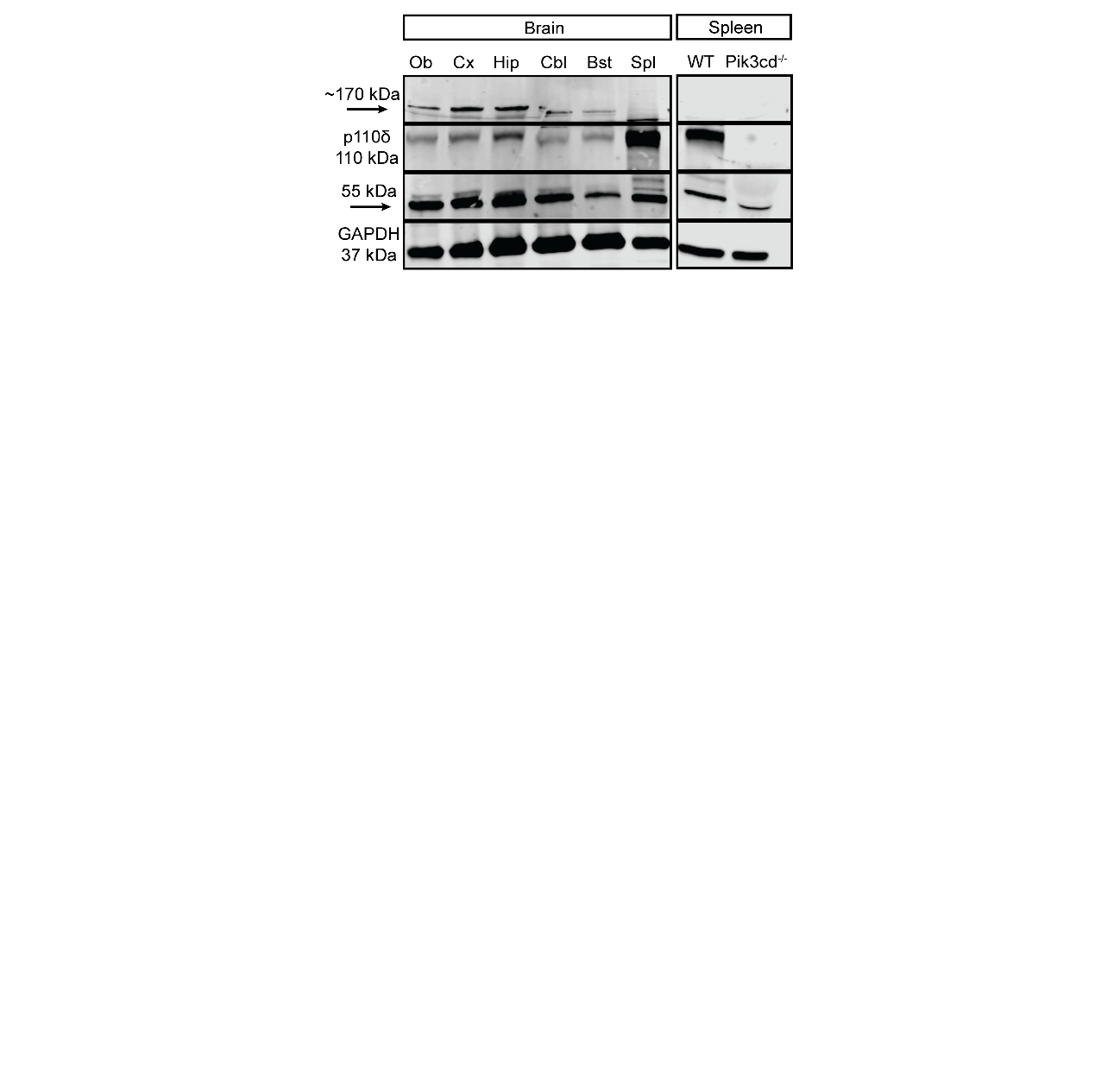
**Figure S2** p110δ expression in brain tissue. Representative blot with the expression of p110δ (110 kDa band) across different brain areas in WT mice. Spleen tissue from WT and p110δ^E1020K^ mice was used as a positive control (Spl) while spleen tissue from a Pik3cd^-/-^ mouse was used as a negative control. GAPDH was used as a loading control. Notice the presence of an antibody unspecific band of 55 kDa, and of brain tissue-specific unspecific bands of approximately 170 kDa. Each section presented (“Brain” and “Spleen”) represents one individual gel. Ob, olfactory bulbs, Cx, cortex, Hip, hippocampi, Cbl, cerebellum, Bst, brainstem, Spl, spleen. Data from one WT mouse is shown


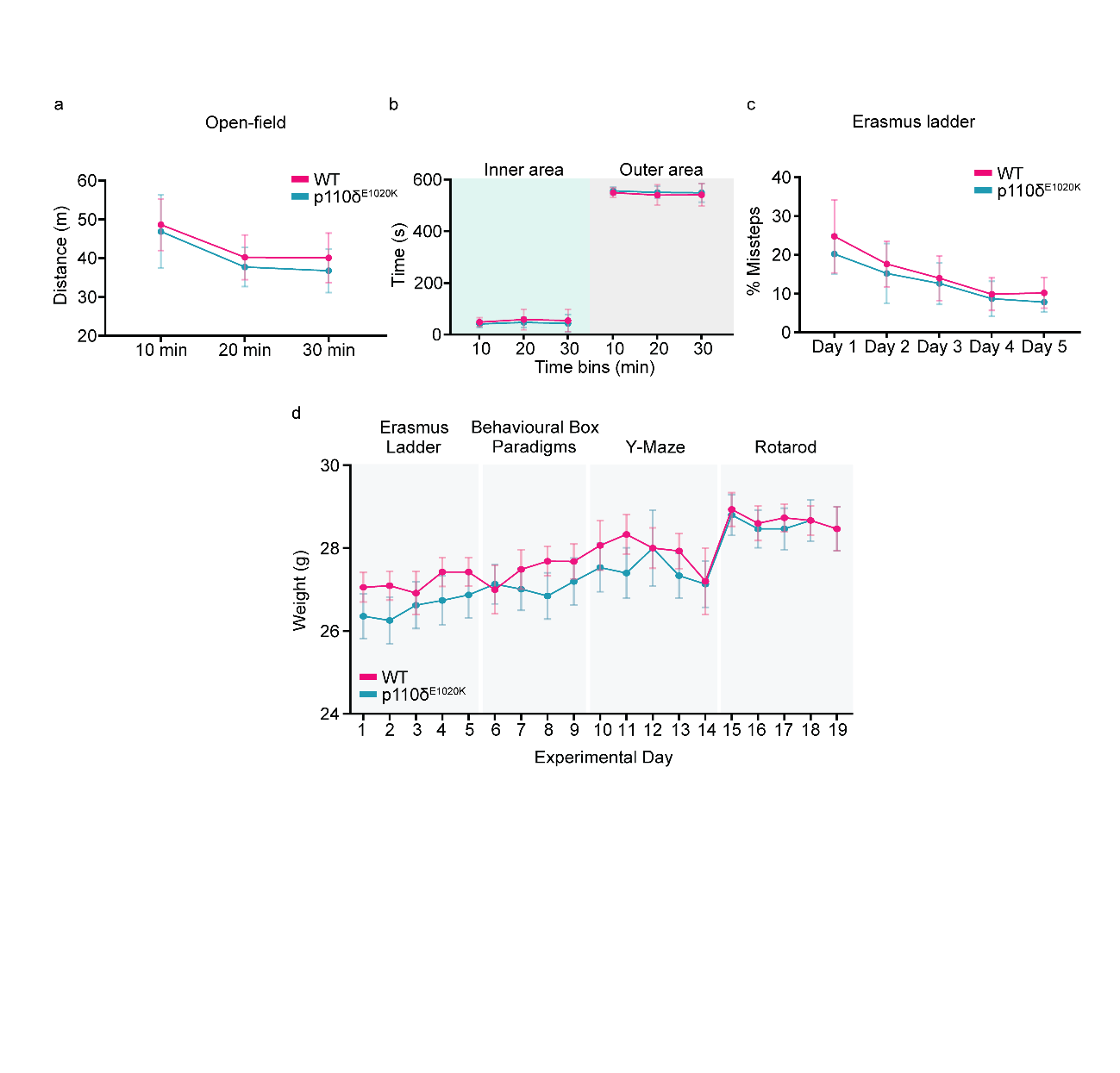


**Figure S3** p110δ^E1020K^ mice do not show changes in gross motor locomotion, anxiety-like behaviours nor weight progression. a) Total distance travelled during OF (mixed effects model), and the total time spent by each mouse in the inner (green) and outer (grey) areas of the OF arena (b) were quantified and binned in 10 minute-periods. c) Average percentage of missteps across the 42 daily trials of the Erasmus ladder (mixed effects model). Data are presented as mean ± SD. n = 15 mice per genotype. d) The average weight, in grams, is presented for both genotypes and for each experimental day. Notice the decrement in weight during the Y-Maze week, likely due to increased physical activity (swimming in the maze). Data is presented as mean ± SEM (mixed effects model). n = 15 mice per genotype, except for day 12 (n = 13 WT and 10 p110δ^E1020K^)


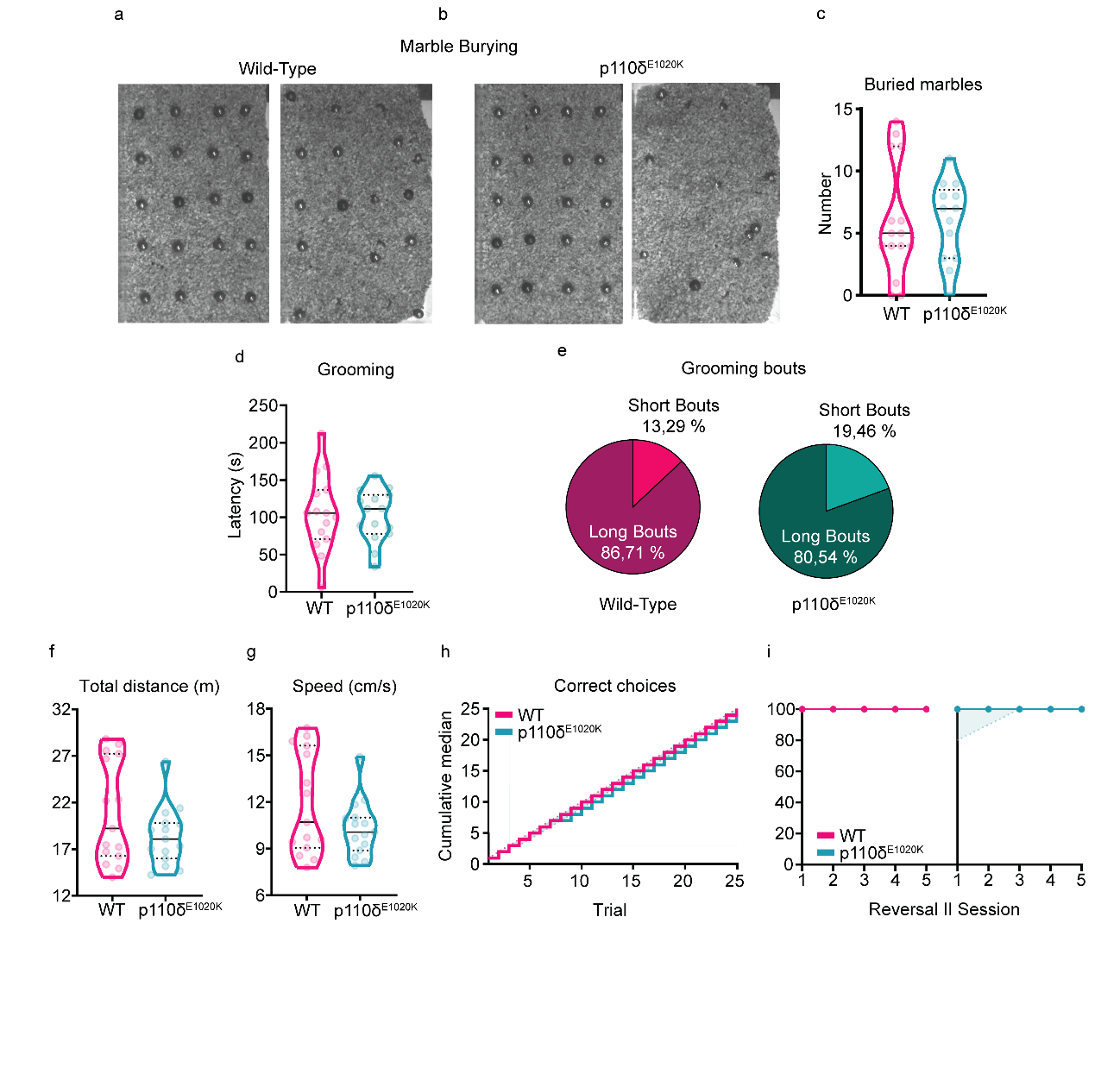


**Figure S4** Repetitive behaviour and swimming analysis in WT and p110δ^E1020K^ mice. a-b) Representative pictures of the 4x5 marble set before (left) and after (right) the 30-min marble burying task. c) Quantification of the total number of buried marbles per mouse, using a 50% buried surface cut-off (2-tailed t-test; data presented as median and quartiles, n = 15 WT and n = 13 p110δ^E1020K^ mice). d) Latency, in seconds, to initiate the first recorded grooming bout (2-tailed t-test; data presented as median and quartiles). e) Average percentage of short (<1 second) and long (>1 second) grooming bouts for WT (left) and p110δ^E1020K^ (right) mice. n = 15 mice per genotype, except for c) (see above). f-g) Total distance swam (f) and average speed (g) of individual mice during the habituation phase of the Y-Maze. h) Step function with the cumulative median of correct arm choices for the reversal II phase. i) Percentage of correct arm choices for each genotype over the five days of reversal II. Data analysed with 2-tailed Mann-Whitney; n = 15 mice per genotype, except for reversal phases where n = 13


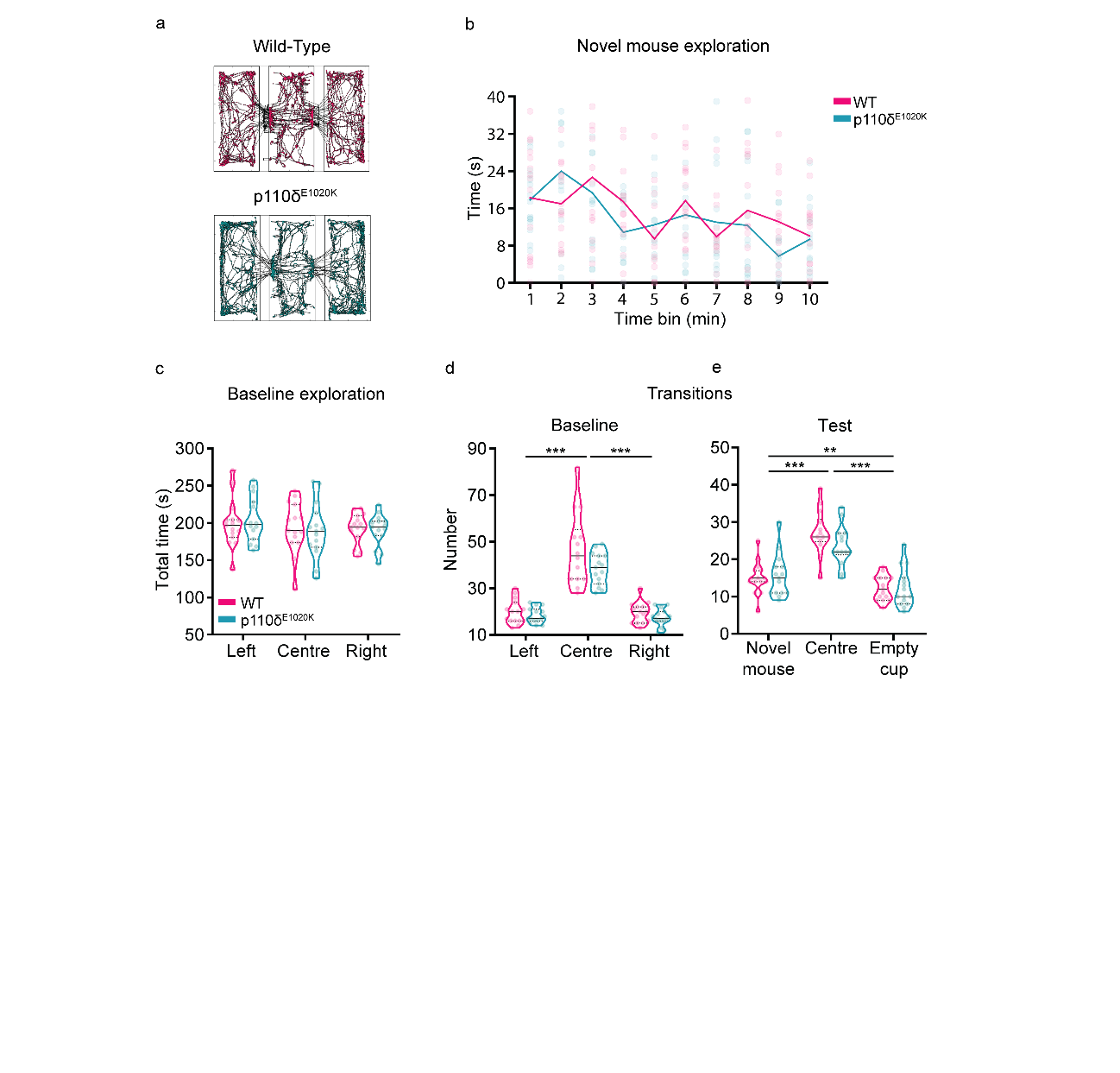


**Figure S5** Social behaviour investigation in p110δ^E1020K^ mice. a) Example of automatically tracked body positions during baseline. b) Total time spent exploring the novel mouse during the 10-minute test phase, split in 1-minute bins (dots represent individual mice and lines connect the mean of each genotype; mixed effects model). c) Total time individual mice spent in each chamber of the SI apparatus during baseline (2-way repeated-measures ANOVA; data presented as median with interquartile range). d-e) Total number of transitions to each chamber made by individual mice during baseline (d) and test (e) (mixed effects model; data presented as median with interquartile range). ** p≤0.01, *** p≤0.001. n = 15 mice per genotype


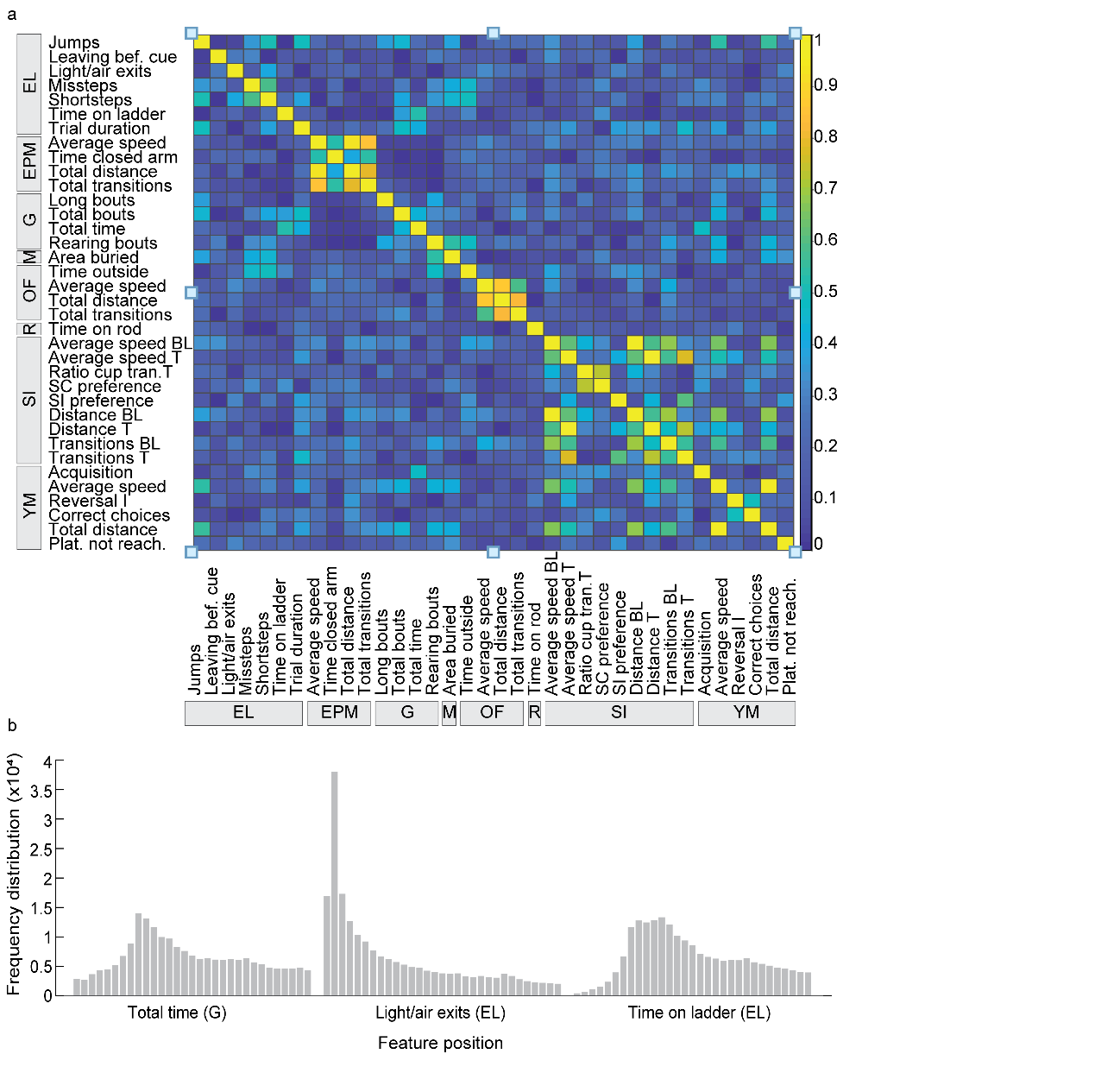


**Figure S6**: LDA correlation matrix and shuffle validation. a) After normal distribution validation, a Pearson’s correlation matrix with all behavioural variables was plotted to identify strongly correlated variables. b) Example of a shuffling trace with the position distribution of the 3 variables that most contribute to LD1. Data was shuffled 200.000 times to achieve an error margin under the 5%, based on the Monte Carlo Simulation concept. Data represents measurements from 15 WT and 15 p110δ^E1020K^ mice. EL, Erasmus ladder, EPM, elevated-plus maze, G, grooming, M, marble burying, OF, open-field, R, rotarod, SI, social interaction, YM, water y-maze
